# Fatty acid esters of hydroxy fatty acids and alkyl-diacylglycerols: minor but bioactive components in human milk

**DOI:** 10.1042/BST20260825

**Published:** 2026-06-08

**Authors:** Megan Li Xian Lee, Untzizu Elejalde, Amaury Cazenave-Gassiot

**Affiliations:** 1 Yong Loo Lin School of Medicine, National University of Singapore, Singapore 117597, Singapore; 2 Singapore Lipidomics Incubator, Life Sciences Institute, National University of Singapore, Singapore 117456, Singapore; 3 Wilmar Innovation Center, Wilmar International HQ, Singapore 138568, Singapore; 4 Department of Biochemistry and CVMD Translational Research Programme, Yong Loo Lin School of Medicine, National University of Singapore, Singapore 117596, Singapore

**Keywords:** Breast milk, ether lipids, FAHFA, lipids, obesity, TG(O)

## Abstract

As the optimal source of nutrition for infants, investigations into the human milk lipidome have been quite extensive. Much of the work, however, has been focused on major lipid components such as triglycerides, possibly undermining its actual complexity. This review focuses on two minor but bioactive lipid classes in human milk: fatty acid esters of hydroxy fatty acids (FAHFAs) and alkyl-diacylglycerols (TG(O)s). FAHFAs are known to exhibit anti-diabetic and anti-inflammatory effects, while TG(O)s are important for the prevention of childhood obesity. With the knowledge that early nutrition and metabolic health influence the risk of metabolic dysfunctions later in life, a comprehensive understanding of FAHFAs and TG(O)s, along with reliable characterisations in human milk, would better allow for the development of accurate human milk fat substitutes. This could have future implications as alternative or preventive treatments for infants with early markers of metabolic dysfunction, including diabetes and obesity. The structural characteristics, pathways for biosynthesis and degradation, bioactivity, dietary sources, and characterisations of FAHFAs and TG(O)s in human milk are discussed. Their statuses as emerging lipid classes, however, is reflected in the incomplete understanding of their biochemical pathways. Characterisations of FAHFAs and TG(O)s in human milk are relatively poor, and contradicting results are reported. This review also addresses the challenges involved in the study of minor lipids in complex biological matrices, and the possible reasons underlying the slower evolution of our understanding of FAHFAs and TG(O)s in human milk and their associations with health outcomes.

## Lipids in human milk

**Figure 1:**
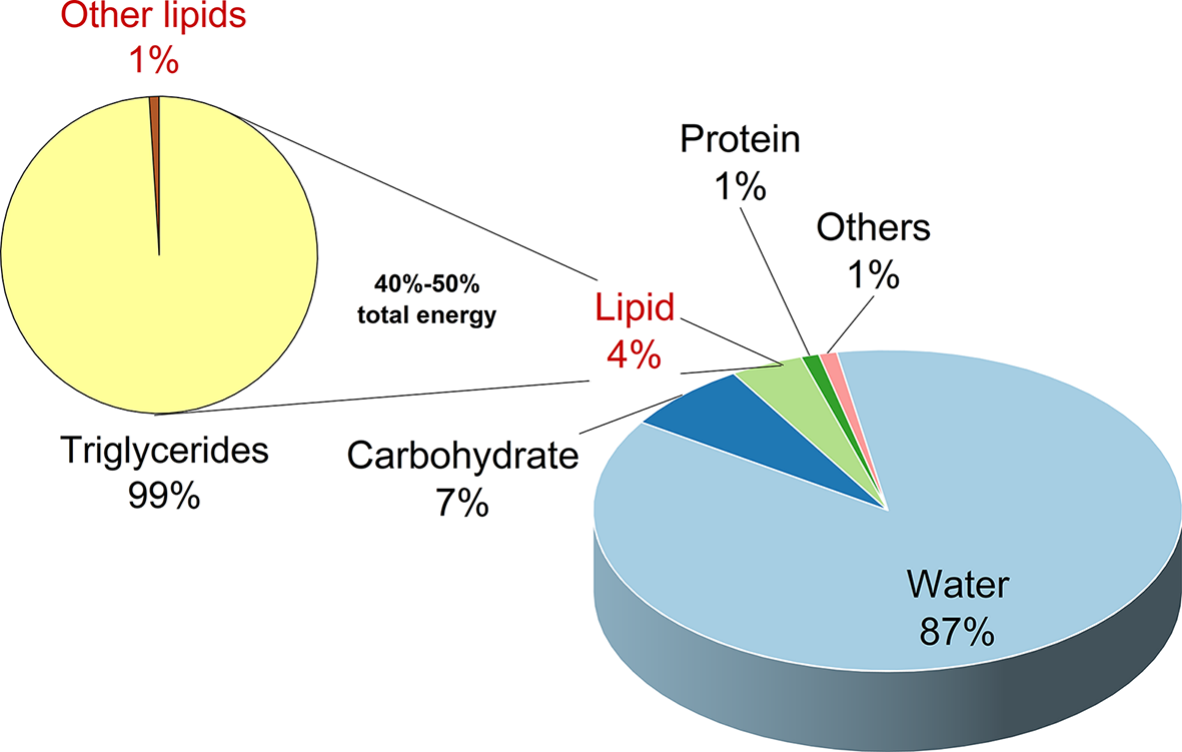
Composition of human milk Composition of human milk. Lipids make up approximately 4% of the milk composition while contributing to 40%–50% of its total energy.

The composition of human milk varies in response to many factors, including temporal variations [1], maternal diet [2,3], obesity [4–6], lactation stages [7], and health status of the mother [8] and nursing infant [9]. Approximately 4% of human milk is composed of lipids, corresponding to 40%–50% of its total energy [10,11] (Figure 1). A vast majority (98%–99%) of these exist as triacylglycerols (a.k.a. triglycerides, TGs) [12–14], and the remainder in the forms of diacylglycerols (DGs), monoacylglycerols, and sterol esters [15]. Polar lipids such as phospholipids are found on the membranes of milk fat globules that surround the TG-rich lipid core [16]. The overwhelming abundance of TGs, however, potentially oversimplifies the actual complexity of the human milk lipidome and creates enormous roadblocks in the study of minor lipid components.

This review focuses on two minor lipid classes that have been found to exert protective effects against diabetes [17–19] and childhood obesity [20,21]: fatty acid esters of hydroxy fatty acids (FAHFAs) and alkyl-diacylglycerols (TG(O)s). Interest in these compounds have arisen due to the growing healthcare burden of common metabolic diseases including diabetes and obesity [22], many of which can develop early in life and extend into adulthood [23,24]. This is further compounded by the fact that early nutrition is known to influence metabolic health later in life [25]. Studies on FAHFAs and TG(O)s in human milk could thus be useful for the development of natural remedies and new treatments against various metabolic diseases during infancy, with potential benefits for long-term public health outcomes. Investigations into the roles of FAHFAs and TG(O)s in human milk, however, remain largely in their infancy. Existing publications tend to focus on profiling the types of FAHFAs and TG(O)s present in human milk, with contradictions and disparities found in current literature. These findings are further discussed later in this review.

## Fatty acid esters of hydroxy fatty acids

### Structural characteristics of FAHFAs

**Figure 2:**
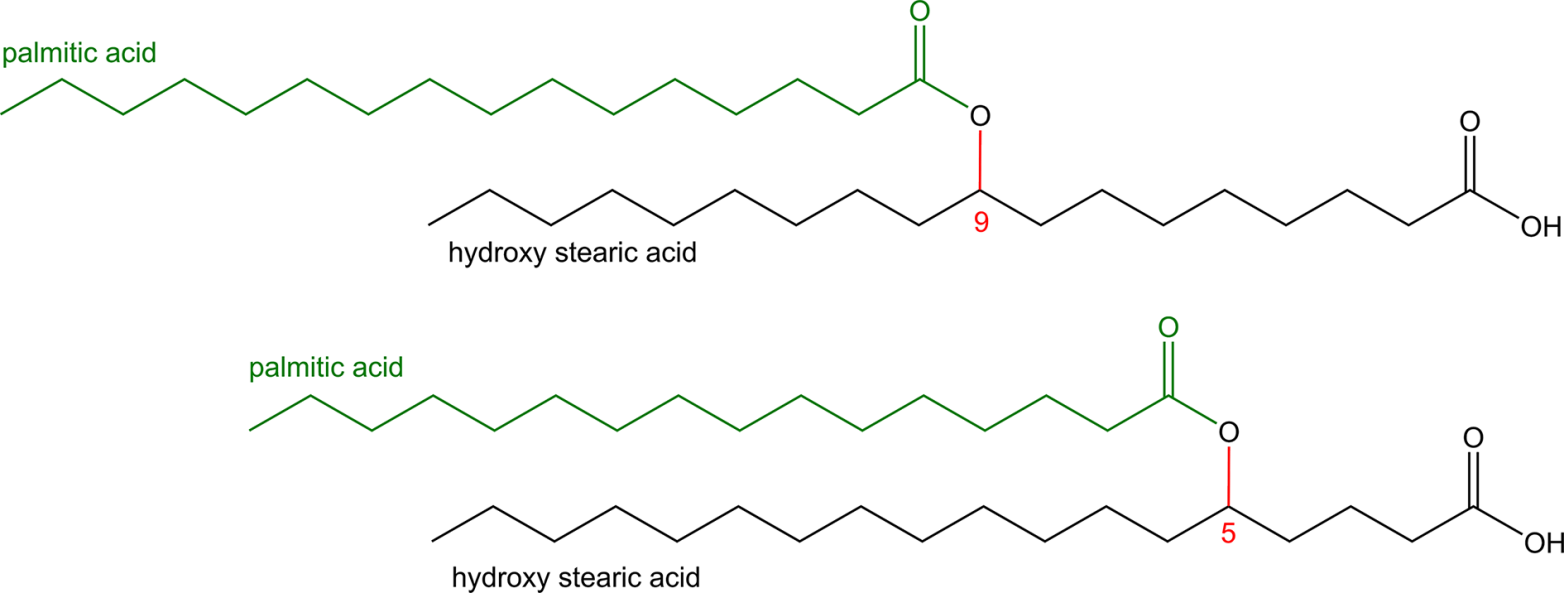
Structures of 9-PAHSA and 5-PAHSA Structures of 9-PAHSA and 5-PAHSA. The palmitic acid chains (green), hydroxy stearic acid chains (black), and positions of esterification (red) are shown.

Branched FAHFAs were initially mentioned as a ‘class of endogenous mammalian lipids with anti-diabetic and anti-inflammatory effects’ in the adipose tissues of mice back in 2014 [19]. FAHFAs can be broadly categorised into two superfamilies: (i) ‘branched’ FAHFAs that are known to regulate metabolism and the immune response, and (ii) ‘linear’ FAHFAs that are mainly found in biosurfactants and the skin barrier [26]. For simplicity, branched FAHFAs will be referred to as ‘FAHFAs’ in subsequent sections. In 2014, mice overexpressing glucose transporter type 4 (GLUT4) were paradoxically found to exhibit increased glucose tolerance despite being obese [19], and lipidomic analysis pointed to the existence of significantly elevated levels of fatty acid (FA) oligomers. Subsequent investigations revealed that these oligomers comprised of a FA coupled to a hydroxy fatty acid (HFA) backbone via an ester bond, earning them the ‘FAHFA’ nomenclature. Regioisomers of FAHFAs vary in their positions of esterification, and these are numerically denoted in their trivial nomenclatures. For example, 9-PAHSA refers to a palmitic acid ester of a hydroxy stearic acid esterified at position 9 relative to the carboxylic acid, while 5-PAHSA refers to esterification at position 5 (Figure 2).

### FAHFA biosynthesis and degradation

In adipose tissues, the biosynthesis of FAHFAs begins with a transacylation reaction for esterification of a FA originating from a TG to an HFA by the adipose TG lipase (ATGL), or from a DG. Overexpression of ATGL was found to increase FAHFA biosynthesis, while inhibition or deletion of the *Atgl* gene inhibited FAHFA biosynthesis [27,28]. An alternative pathway of FAHFA synthesis could possibly involve the activity of peroxiredoxin 6 (Prdx6), a multifunctional enzyme with phospholipase A_2_, peroxidase, and acyltransferase activity. Mutation of Prdx6 C47S was found to reduce FAHFA levels by impairing its peroxidase activity while Prdx6 deletion was found to negatively affect FAHFA regioisomer abundance, especially for FAHFAs with hydroxy groups farther from the HFA α carbon [29].

FAHFAs are hydrolysed into FAs and HFAs by carboxyl ester lipase (CEL) [30], androgen-induced gene 1 (AIG1), androgen-dependent tissue factor pathway inhibitor (TFPI)-regulating protein (ADTRP) [31], or hormone-sensitive lipase (HSL) [28,32]. Of these, CEL has been documented in human milk [33,34].

**Figure 3:**
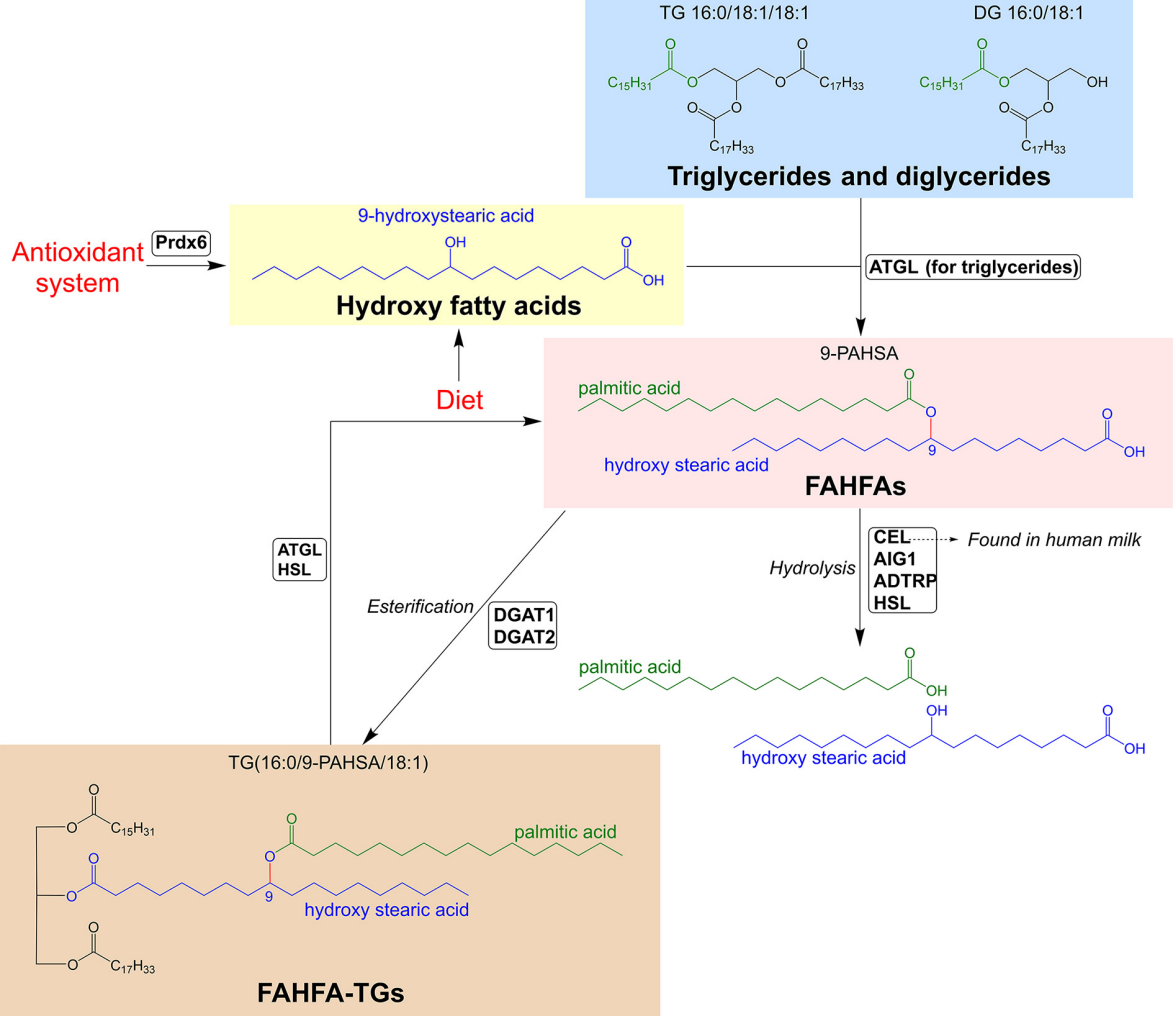
Pathways for the biosynthesis and degradation of FAHFAs Pathways for the biosynthesis and degradation of FAHFAs. The FA portions are coloured green, while the HFA portions are blue. FAs from TGs/DGs are esterified to HFA chains. These FAHFAs can be hydrolysed back into their FA and HFA chains via the activity of CEL, AIG1, ADTRP, or HSL. CEL has been documented in human milk. Alternatively, FAHFAs can be stored as FAHFA–TGs after esterification by DGAT1 and DGAT2. The activity of ATGL and HSL then releases FAHFAs from FAHFA-TGs for subsequent reactions.

FAHFAs can be esterified onto the glycerol backbone of TGs to form FAHFA-TGs, the major reservoir for FAHFAs. This occurs by enzymatic activity of diglyceride acyltransferases (DGAT1 and DGAT2) and other alternative mechanisms that have yet to be discovered [35]. FAHFA-TGs are then catalysed by ATGL and HSL [32] to release FAHFAs for downstream reactions. In human milk, the breast white adipose tissues that are transdifferentiated into the milk glands are possible sources of free FAHFAs and FAHFA-TGs [36,37]. The pathways for the biosynthesis and degradation of FAHFAs have been illustrated in Figure 3.

### Bioactivity of FAHFAs

Levels of FAHFAs vary with different pathophysiological states and exert anti-diabetic and anti-inflammatory effects. For example, insulin-resistant individuals have been found with lower levels of PAHSAs in both serum and adipose tissues [19]. This is consistent with the fact that PAHSAs are known to enhance insulin-stimulated glucose uptake in glycolytic muscles and hearts, enhance hepatic insulin sensitivity [38], improve glucose-stimulated insulin secretion in islets [17,28], and suppress endogenous glucose production by enhancing insulin activity [38].

In white adipocytes, PAHSAs enhance insulin-mediated suppression of lipolysis and lipid metabolism while improving glucose tolerance via the activities of 5- and 9-PAHSA. 9-PAHSA, for example, is an agonist of the G protein-coupled receptor (GPCR) GPR120 [19,39]. Activation of GPR120 triggers downstream signalling pathways, including the phosphoinositide 3-kinase (PI3K)/protein kinase B Akt pathway, which enhances the translocation of GLUT4 to the plasma membrane to enhance glucose uptake and increase glucose tolerance [40,41]. Both 5- and 9-PAHSA promote insulin-mediated suppression of lipolysis and suppression of glucose production by increasing sensitivity to insulin and regulating the amount of free FAs released into the bloodstream [38,42].

In pancreatic β-cells, PAHSA binds to GPR40 to promote the release of insulin via cell signalling pathways. The activity of PAHSA on GPR40 might be similar to that of free FAs where phospholipase C (PLC) is activated and phosphatidylinositol-4,5-biphosphate (PIP_2_) is hydrolysed. This results in the formation of DG and inositol-1,4,5-triphosphate (IP_3_). IP_3_ triggers the release of calcium from the endoplasmic reticulum (ER) and increases cytosolic calcium ion levels, while DG promotes the activation of protein kinase D1 (PKD1), ultimately potentiating insulin granule exocytosis [17,43]. The anti-diabetic effects of PAHSAs are further evidenced by the protective effects of 5- and 9-PAHSA supplementation on β-cell function and survival. These protective effects could be attributed to a reduction in ER stress and mitogen-activated protein kinase (MAPK) signalling in non-obese diabetic mice, resulting in the delay and/or reducing the risks of developing type 1 diabetes [18]. This mechanism is mediated through the glucagon-like peptide 1 receptor (GLP-1R). More recently, it has been discovered that 5- and 9-PAHSA could also exert a protective effect on pancreatic β-cells by reducing cellular senescence. This is achieved by increasing the expression of murine double minute 2 (Mdm2), a negative regulator of the p53 tumour suppressor [44].

In terms of cardiovascular health, 9-PAHSA has been found to reduce microvascular damage acquired during cardiac ischaemia/reperfusion injury by activating autophagy via the liver kinase B1/adenosine monophosphate (AMP)-activated protein kinase/Unc-51-like kinase 1 (LKB1/AMPK/ULK1) pathway. This promotes the degradation of the stimulator of interferon genes (STING) and the survival of coronary artery endothelial cells/cardiac microvascular endothelial cells (CMECs) against ischaemia/reperfusion injury [45].

**Figure 4:**
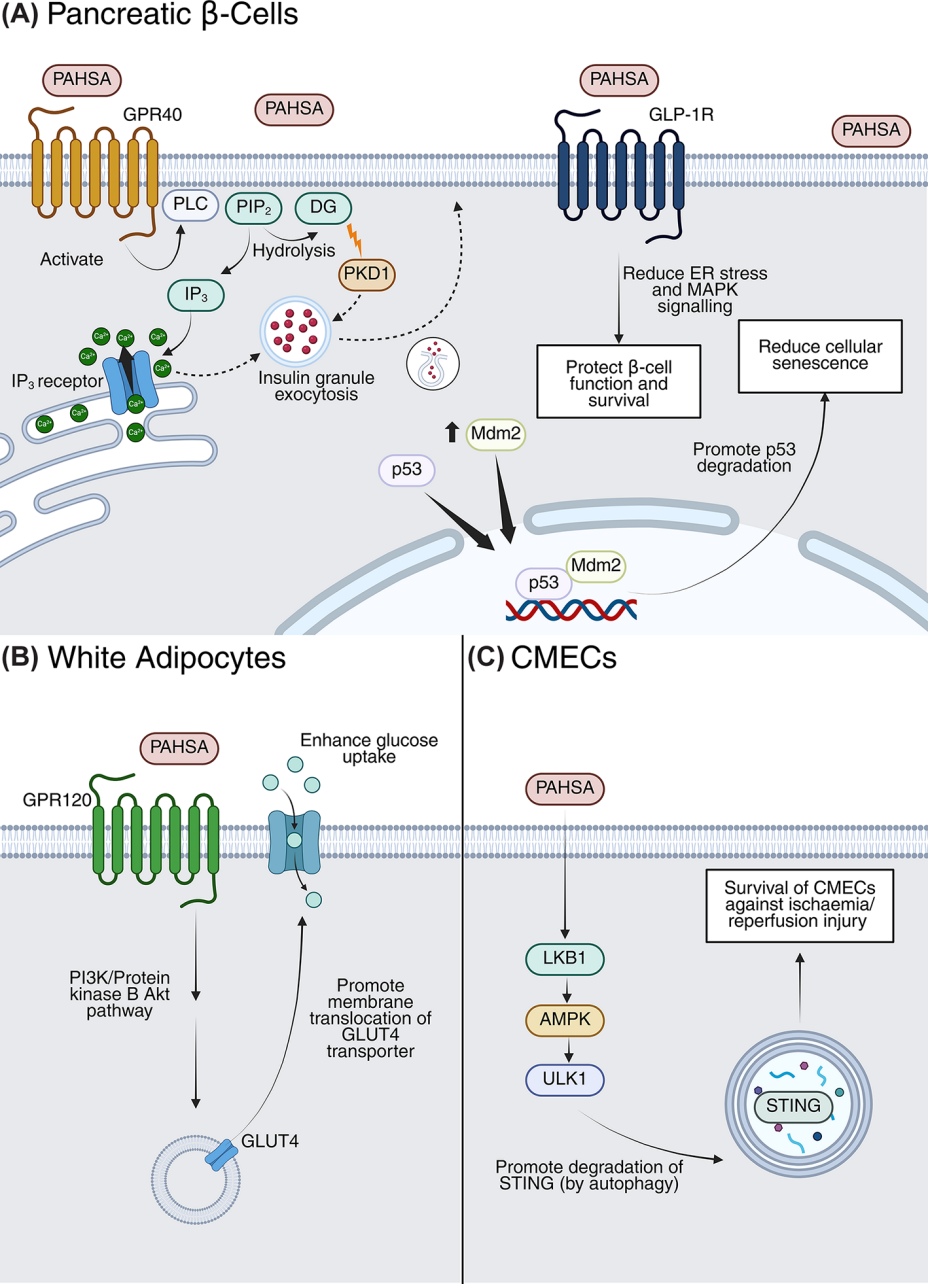
Bioactivity of PAHSA in different cell types Bioactivity of PAHSA in (**A**) pancreatic β-cells, (**B**) white adipocytes, and (**C**) CMECs. (**A**) In pancreatic β-cells, PAHSA binds to GPR40 and activates PLC. PIP_2_ is hydrolysed to form DG and IP_3_. IP_3_ triggers the release of calcium ions from the ER, while DG promotes the activation of PKD1. These cumulate in the potentiation of insulin granule exocytosis. PAHSA can also bind to GLP-1R, reducing ER stress and MAPK signalling to enhance protection of β-cell function and survival. PAHSA also increases the expression of Mdm2, a negative regulator of p53, and suppresses cellular senescence. (B) In white adipocytes, PAHSA binds to and activates the GPR120-mediated PI3K/protein kinase B Akt pathway. This promotes membrane translocation of the GLUT4 transporter and enhances glucose uptake and increases glucose tolerance. (**C**) PAHSA also plays a role in activating autophagy via the LKB1/AMPK/ULK1 pathway and promotes the degradation of STING for the survival of CMECs against ischaemia/reperfusion injury.

The bioactivity of PAHSAs in white adipocytes, pancreatic β-cells, and CMECs has been summarised in Figure 4.

### Dietary sources of FAHFAs

FAHFAs have been found in numerous fruits, vegetables, food of animal origin, as well as human milk. Naturally occurring FAHFAs, for example, have been found in various types of teas that increase with the degree of fermentation. A previous study on sixteen types of tea, including four post-fermented teas, was conducted to profile FAHFA diversity via 2-dimethylaminoethylamine (DMED)-assisted labelling. High levels of FAHFA were found in Liubao and Fu brick teas, while lower levels of FAHFAs were found in green, white, and oolong teas [46]. Apart from tea, edible vegetable oils are another source of FAHFAs. Rice bran oil (6.12 μg/g), avocado oil (2.45 μg/g) and sesame oil (2.04 μg/g) are sources of FAHFAs, with oleic acid ester of hydroxy stearic acid (OAHSA) as the predominant FAHFA quantified [47]. Fruit sources of FAHFA include clementine (0.25 μg/g), pineapple (0.22 μg/g), and strawberries (0.16 μg/g) [48].

Commonly consumed foods of animal origin such as beef, chicken, and eggs are also viable sources of FAHFA. Beef, for example, has been found to contain approximately 9.16 ng/g of PAHSA, while egg yolks were found to contain approximately 32 ng/g of PAHSA [19].

### Characterisation of FAHFAs in human milk

Hundreds of FAHFA species have been reported in human milk. A comprehensive list of reported putative species has been summarised in Supplementary Table S1. In terms of specific FAHFA molecular species, PAHSA has been found to decrease in human milk from nursing women with obesity [49]. A contradicting report, however, has demonstrated that obese/overweight nursing women produce human milk with higher concentrations of 5-PAHSA [50]. Large differences in the quantitation of FAHFAs in existing literature have also been reported. FAHFA 16:0/9O(FA16:0) collected within 72 hours postpartum, for example, has been reported at concentrations of 0.09 nmol/l in one publication [37], and up to 19.2 nmol/l in another [50]. This further contributes to uncertainties in characterisations of FAHFAs in human milk and conclusions on their associations with health outcomes.

Some studies have also characterised FAHFAs as part of the whole human milk lipidome, and associations have been found with childhood adiposity rebound [51], gestational diabetes mellitus (GDM) status [52], and gestational ages [53]. FAHFAs in human milk, specifically FAHFA 18:0/16:1;O and FAHFA 18:0/18:1;O, for example, were found to be positively associated with childhood adiposity rebound for exclusively breastfed infants [51]. The authors, however, acknowledged limitations in available literature that impede further discussions on the possible reasons for this phenomenon. In terms of infant bodyweight gain, increase in length, and growth of head circumferences, analysis of colostrum found negative associations with FAHFA 18:1/20:3;O and FAHFA 18:2/20:4;O at birth, and diminishing associations from 42 days to 3 months postpartum [52]. Similar limitations were encountered in rationalising these results beyond hypothesising that adaptations in the nutritional composition of human milk could reflect specific needs of each infant.

Upregulations of FAHFAs such as FAHFA 2:0/17:2;O, FAHFA 12:0/18:2;O, FAHFA 16:1/22:4;O, FAHFA 18:1/18:2;O, FAHFA 18:2/14:1;O, FAHFA 20:2/20:1;O, FAHFA 20:3/22:5;O, and FAHFA 22:3/22:4;O were also observed in the mature milk of preterm infants compared to full-term infants [53]. Given that adipose tissues are one of the sites for FAHFA biosynthesis, the authors proposed that this could be attributed to the higher proportion of adipose tissues in the breasts of mothers with preterm infants compared to full-term infants [53]. While this could be reflective of the roles FAHFAs play in the maturation of preterm infants, further studies are required to better establish the bioactivity of FAHFAs in human milk.

In a more recent study on the colostrum, transitional, and mature milk from healthy Chinese mothers, 71 FAHFA families and 338 regioisomers were identified. The concentrations of FAHFAs were generally found to decrease with lactation [54]. 5-PAHSA, however, was largely undetectable, and comparisons to previous studies were thus not made [49,50]. Profiling of FAHFA regioisomers was conducted based on chromatographic separations, an approach that has not been widely adopted due to difficulties in achieving sufficient resolution. A similarly recent study on mature milk from Singapore identified 16 FAHFA precursors and 36 unique molecular species [55], indicative of wide discrepancies in the results from existing literature.

## Alkyl-diacylglycerols

### Structural characteristics of TG(O)s

**Figure 5:**
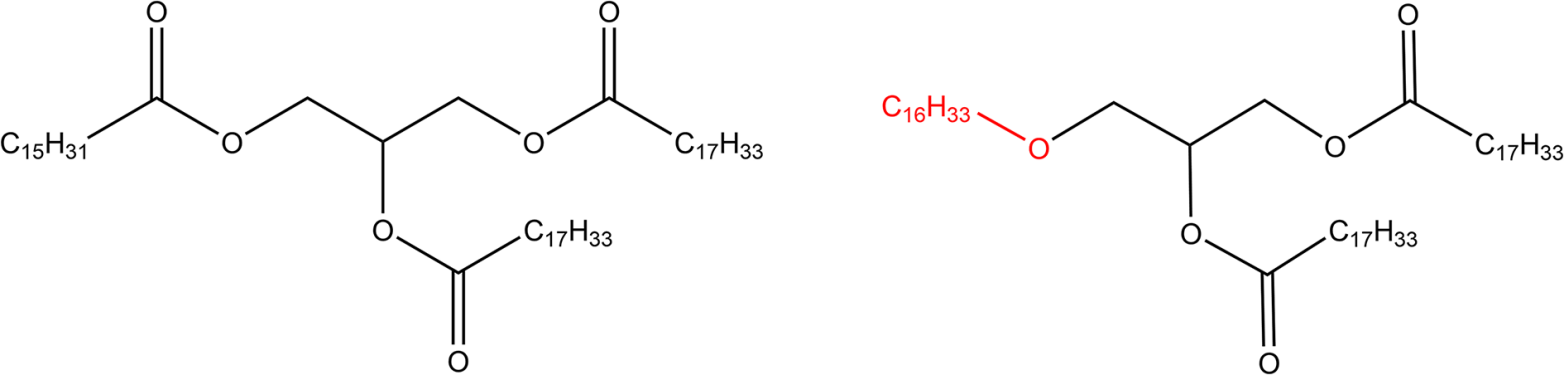
Structures of 1-palmitoyl-2-oleoyl-3-oleoylglycerol and 1-O-palmitoyl-2-oleoyl-3-oleoylglycerol Structures of 1-palmitoyl-2-oleoyl-3-oleoylglycerol and 1-O-palmitoyl-2-oleoyl-3-oleoylglycerol. The alkyl chain has been highlighted in red.

Unlike FAHFAs, TG(O)s are structurally similar to TGs, except for an ether linkage instead of an ester bond at the *sn*-1 position (Figure 5). Following the LIPID MAPS naming convention of glycerol lipids, TG(O)s can be presented at the species level (TG O-52:2), molecular species level where chain positions are unknown (TG O-16:0_18:1_18:1), *sn*-position level (TG O-16:0/18:1/18:1), and the full structural level (TG O-16:0/18:1(9Z)/18:1(11Z)) [56]. TG(O)s have been documented in marine creatures such as deep-sea corals [57], shark livers [58,59], and squids [60], possibly for energy storage. In humans, TG(O)s have been reported in plasma [61] and human milk [55,62–64]. For simplicity, the O-alkyl chain is subsequently referred to as the ‘alkyl chain’.

### TG(O) biosynthesis and degradation

Acyltransferases are known to be involved in the synthesis of TG(O)s via a two-step acylation reaction. It has been proposed that the first acylation takes place on 1-monoalkylglycerol (1-MAkG), producing a monoalkyl-monoacylglycerol with a fatty acyl tail at the *sn-*2 or *sn-*3 position. A final acylation step then produces a TG(O) [65]. Various acyltransferases were screened in the present study, identifying DGAT1, DGAT2, monoacylglycerol acyltransferase (MGAT)2, MGAT3, acyl coenzyme A (acyl-CoA):wax-alcohol acyltransferase 2/multifunctional O-acyltransferase (MFAT), and DGAT candidate 3 as possible enzymes responsible for both acylation steps. Studies on the adrenal gland of DGAT1 knock-out mice presented with reduced levels of TG(O), suggesting that DGAT1 plays a role in TG(O) synthesis in the adrenal glands.

Alkyl-lysophosphatidic acid (alkyl-LPA) can also be obtained from the diet or converted from 1-MAkG in the presence of an alkyl kinase. Acyltransferases will then convert alkyl-LPA to alkyl-phosphatidic acid (alkyl-PA). A phosphatidate phosphatase acts on alkyl-PA and catalyses the dephosphorylation of alkyl-PA into a plasmanyl DG (monoalkyl-monoacylglycerol). TG(O) is eventually produced after acylation of monoalkyl-monoacylglycerol [66–69].

TG(O)s may be broken down by lipases back into monoalkyl-monoacylglycerols and MAkGs, thereby serving as an alkylglycerol pool for the synthesis of other ether lipids, including plasmalogens [59,65].

**Figure 6:**
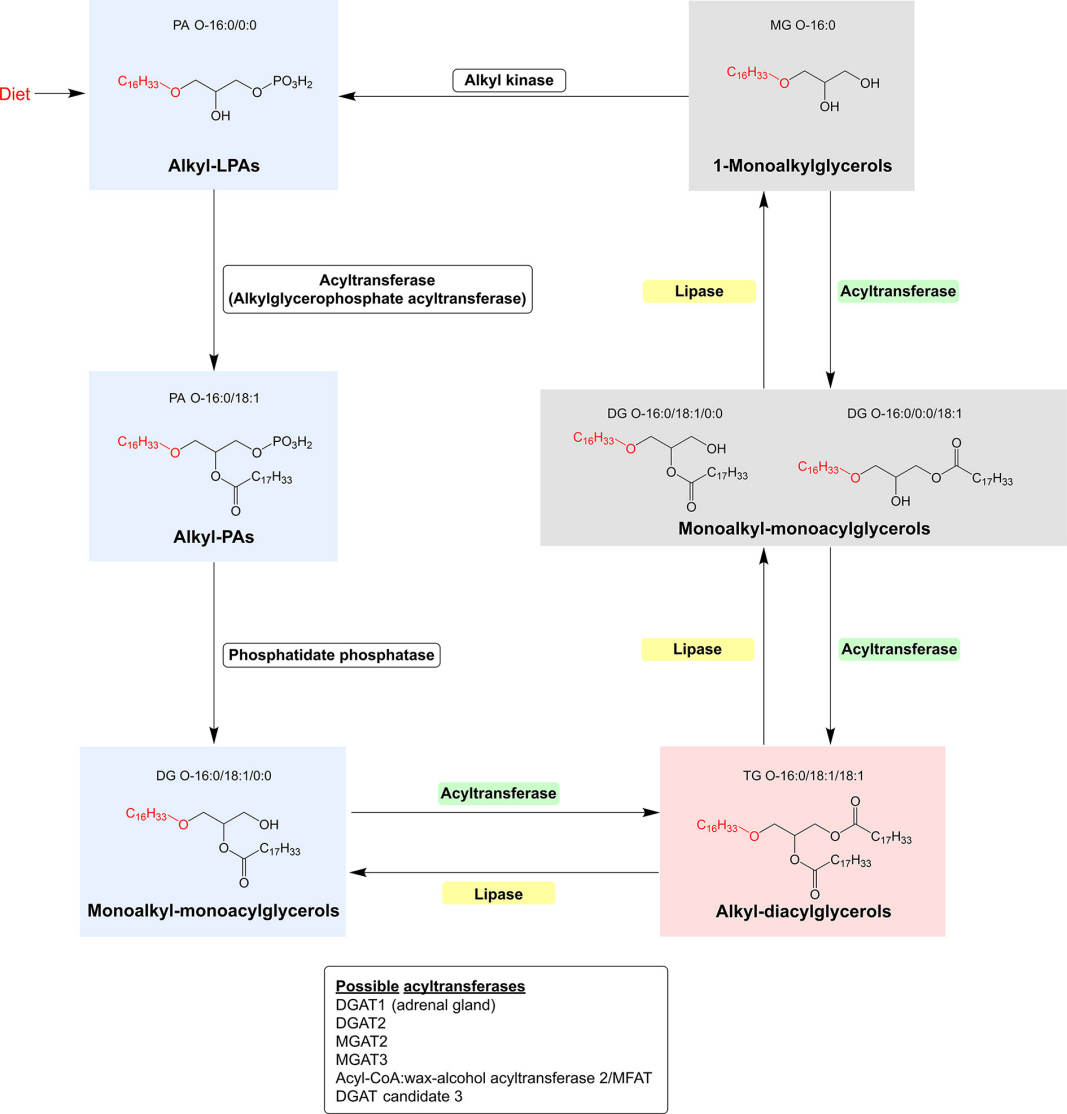
Pathways for the biosynthesis and degradation of TG(O)s Pathways for the biosynthesis and degradation of TG(O)s. The alkyl chain has been highlighted in red. In the first pathway, acylation occurs on 1-MAkGs to produce monoalkyl-monoacylglycerols. The next acylation then converts these into TG(O)s. The alternate pathway involves the acylation of alkyl-LPAs obtained from the diet or produced from 1-MAkG in the presence of an alkyl kinase. Alkyl-LPAs are then converted to alkyl-PAs by acyltransferases, and by phosphatidate phosphatases to monoalkyl-monoacylglycerols. Similarly, the next acylation then converts these into TG(O)s. TG(O)s can be broken down into monoalkyl-monoacylglycerols and 1-MAkGs by lipases.

Pathways for the synthesis and degradation of TG(O)s have been illustrated in Figure 6.

### Bioactivity of TG(O)s

**Figure 7:**
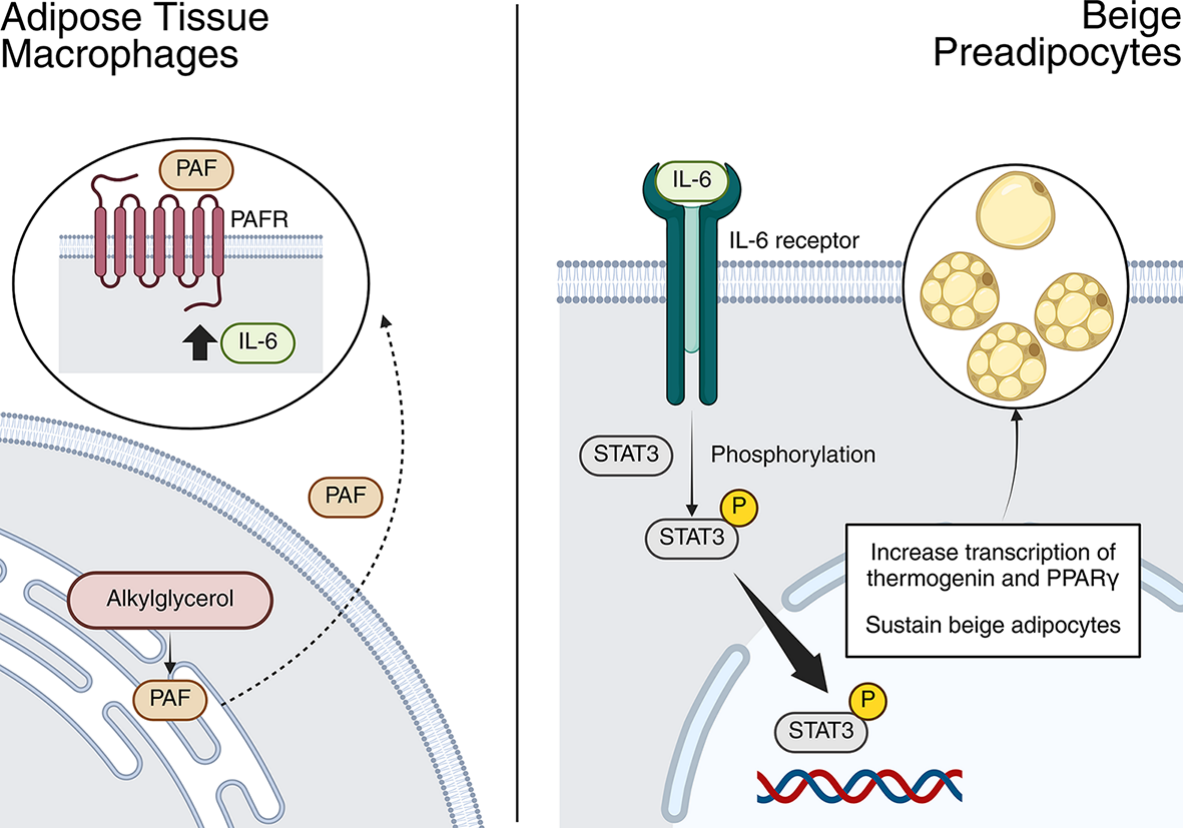
Roles of TG(O)s in sustaining beige adipocytes Role of TG(O)s (human milk alkylglycerols) in sustaining beige adipocytes. In ATMs, alkylglycerols can be metabolised into PAFs that subsequently bind to PAFRs and increase the expression and release of IL-6. IL-6 then phosphorylates STAT3 to increase the transcription of thermogenin and PPARγ, sustaining the pool of beige adipocytes and preventing premature transition of beige preadipocytes into white adipocytes.

Despite mentions of their observations in plasma [61] and adipose tissues [21], studies on TG(O)s in humans remain limited. They are known to be important for the prevention of childhood obesity by promoting the development of beige adipocytes in infants [20,21]. This is thought to occur through adipose tissue macrophage (ATM) signalling. With TG(O)s as the primary ether lipid in human milk [21], human milk alkylglycerols are first metabolised in ATMs into the platelet activating factor (PAF), which interacts with the platelet activating factor receptor (PAFR), increasing the expression and release of interleukin-6 (IL-6) [20,70]. IL-6 could then phosphorylate signal transducer and activator of transcription 3 (STAT3) at tyrosine 705 (Tyr705) in beige preadipocytes, enhancing transcription of various genes including thermogenin and peroxisome proliferator-activated receptor gamma (PPARγ) [20,71,72]. This promotes the differentiation of the preadipocytes into beige adipocytes and prevents premature transition of these preadipocytes into white adipocytes (Figure 7). This TG(O)-activated signalling, however, occurs only during infancy and becomes increasingly inactivated towards adulthood [20].

With TG(O)s functioning as alkylglycerol reservoirs for the synthesis of ether lipids, other functions of TG(O)s tend to be attributed to their bioactivity in the form of alkylglycerols. These include anti-obesity effects beyond infancy and improving insulin resistance.

Supplementation of plasmalogens to mice with adipose-specific peroxisomal biogenesis factor Pex16 knockout (*Pex16-AKO*), for example, was found to rescue mice experiencing diet-induced obesity by increasing mitochondrial copy number, improving mitochondrial function, and rescuing thermogenesis [73]. A subsequent study found that a transmembrane protein important for regulating mitochondrial fission and fusion, TMEM135 [74], showed the most significant decrease in mitochondria from the beige adipose tissues of *Pex16-AKO* mice. This adds further credence to the importance of alkylglycerols in energy homeostasis [75]. The beneficial effects of alkylglycerols in obese individuals could also be attributed to their anti-inflammatory responses. Alkylglycerol supplementation, for example, was found to reduce levels of inflammatory cytokines such as tumour necrosis factor, TNF-α [76], a chronically elevated compound in obesity identified as a potential target for the treatment of obesity-linked metabolic disorders such as insulin resistance [77,78].

The effects of alkylglycerols on insulin resistance, however, presents some challenges. Different alkylglycerol isoforms have been observed to exert different effects on obese mice fed with a high-fat diet. Selachyl alcohol (1-O-octadec-9-enylglycerol), for example, was found to reduce fasting glucose levels and insulin levels while batyl alcohol (1-O-octadecylglycerol) was found to increase fasting insulin levels [79]. Additional studies are thus required for a more comprehensive understanding about the effects of individual alkylglycerols on insulin resistance.

### Dietary sources of TG(O)s

Marine organisms, especially shark livers, are a common source of TG(O)s. Approximately 18% of squalene-free shark liver oil, for example, is composed of TG(O)s [80]. Its touted effects against obesity-related metabolic diseases [59], regulation of immunomodulatory and inflammatory pathways, and anti-tumour activity [81,82] have cemented the use of shark liver oil as a folk remedy for various ailments. This includes immunomodulation in geriatric patients before surgery [83], maintenance of good vascular health [84], and as an adjunct treatment for cancers [85], although its alleged effectiveness as a natural remedy are not specific to TG(O)s.

A study from the 1970s reported on the presence of TG(O)s in teleost fish [86]. However, there has since been a gap in the identification and quantitation of TG(O)s in edible fishes and we are thus unable to conclude if the intake of fish contributes to a substantial amount of dietary TG(O)s. Antarctic krill oil has also been identified as a potential source of dietary TG(O)s mainly in the forms of TG O-50:2, TG O-48:1, TG O-48:2, TG O-46:1, and TG O-52:2 [63].

### Characterisation of TG(O)s in human milk

Measurements of total TG(O)s in human milk are typically based on whole lipid extractions [64] or the measurement of alkylglycerols after saponification [62,87]. An earlier study on lipids in human milk collected at different timepoints starting from the colostrum to mature milk has demonstrated a reduction in TG O-52:2, TG O-52:1, and TG O-50:1 [64]. The reason for this phenomenon remains unknown beyond lipids in human milk adapting to the changing nutritional needs of breastfed infants.

In a more recent approach, whole lipid extraction with butanol and methanol was used for the analysis and identification of TG(O)s from human milk, formula milk, and animal milk. The analysis of alkylglycerols was used to quantify total TG(O)s [62]. Researchers found TG O-52:2 to be present in highest abundance, followed by TG O-52:1 and TG O-50:1. The present study has shown that bovine, goat, and soy-based formula milk contain significantly lower levels of ether lipids (including TG(O)s) compared to human milk (*p* < 0.001). This finding comes in spite of the fact that total lipid concentrations were similar between human milk and formula milk where TGs formed a large majority of the lipid content [62]. It is thus evident that while formula milk has been produced as a human milk substitute, it remains a poor reflection of the actual complexity of human milk. This is of special concern for minor but bioactive lipids, including TG(O)s, because these minor lipid classes could be important for biological processes that have yet to be established. It could also possibly help to explain the prevalence of childhood obesity in formula-fed infants compared to breastfed infants [88], and perhaps even the reduction of inflammation associated with breastfeeding [89].

In yet another study that characterised TG(O)s from 6 healthy mothers in a Chinese hospital, slightly different results were obtained. No other demographic information, however, was available. TG O-52:2 remained present in highest abundance, followed by TG O-52:3 and TG O-52:1 [63]. With the aim of developing a new method for identifying alkylglycerols in food, the characterisation of TG(O)s in human milk was not the focus of the study, possibly explaining its small sample size. A recent study utilising solid phase extraction (SPE) for the isolation of TG(O)s and removal of TG contaminants in another small Singapore-based cohort has also been reported, identifying 19 TG(O) precursors and 46 molecular species [55].

A list of TG(O)s reported in human milk has been summarised in Supplementary Table S2. The scarcity of available studies on TG(O)s in human milk could be reflective of the challenges associated with the characterisation of these minor compounds in complex, TG-rich matrices. This is especially difficult due to the similar polarities and molecular masses between TG(O)s and TGs, resulting in overlapping chromatograms during liquid chromatography–mass spectrometry (LC–MS) analysis dominated by the highly abundant TGs. Current methods for the extraction and analysis of TG(O)s (and FAHFAs), along with existing challenges, are further elaborated in the next section.

## Study of minor lipids in human milk

While there has been growing interest in the characterisation of minor lipids, including FAHFAs and TG(O)s, in human milk, these studies are often challenged by the low abundances and need for sensitive techniques, both of which contribute to difficulties in collecting reproducible and reliable results. While well-established methods including Folch [90], Bligh and Dyer [91], and methyl-tert-butyl ether (MTBE) [92] extractions are available, these methods are used to obtain whole lipid extracts and might not be suitable for the extraction of minor lipids without further modifications [93,94].

Interferences from major lipid components further complicate the extraction process, often resulting in the need for further analyte isolation, purification, and concentration via various processes such as SPE. The addition of SPE, however, is not without its disadvantages. The use of silica cartridges, for example, often result in reduced retention and low reproducibility because ‘their hydrogen binding sites are mainly destroyed by water’ [95]. Systematic validations should thus be conducted to ensure that results obtained are reproducible, with careful selections of elution solvents, optimisations of elution volumes, and selection of appropriate stationary phases.

In this section, we will focus on some of the existing methods and challenges specific to the study of FAHFAs and TG(O)s.

### Methods for the extraction and analysis of FAHFAs

FAHFAs are typically extracted using liquid–liquid extraction techniques such as the Folch method of extraction and the Bligh and Dyer method of extraction. Acidification during the extraction process has proven useful in the recovery of FAHFAs [96], although SPE remains important for the enrichment of FAHFAs from various tissues [96,97]. FAHFAs are usually eluted from silica SPE cartridges with ethyl acetate for subsequent analysis [96,97]. The LC–MS analysis of FAHFAs typically involves compound separation on a C18 column followed by mass spectrometry analysis in negative ionisation [19,98]. This method has also been modified and validated in human milk samples [55]. More recently, derivatisation-based methods such as DMED/d_4_-DMED labelling have been used to improve sensitivity and selectivity [54,99–101], followed by analysis in positive ionisation. However, heat is usually applied during derivatisation and might cause degradation of some analytes of interest.

In terms of mass spectrometry analysis, multiple reaction monitoring (MRM) [47,102] and sequential window acquisition of all theoretical fragment ion spectra mass spectrometry (SWATH MS) [101,103] have been used for the analysis of FAHFA, along with Orbitrap and Orbitrap hybrid technologies [104] for MS^2^ determination of corresponding FA and HFA groups.

### Methods for the extraction and analysis of TG(O)s

Similar to that of FAHFAs, the extraction of TG(O)s typically begins with whole lipid extraction via liquid–liquid techniques such as Folch extraction [57,90], Bligh and Dyer [91,105], and the MTBE method of extraction [65,92]. Subsequent steps for the isolation of TG(O)s, however, vary widely.

Thin layer chromatography (TLC), for example, can be used as part of TG(O) isolation and analysis. In some cases, TLC was used to isolate TG(O)s and lipid analysis was conducted based on the images of lipid bands obtained [65]. Alternatively, some researchers have adopted the approach of reducing whole lipid fractions after extraction to produce alkylglycerols from TG(O)s. SPE can then be used to isolate alkylglycerols with quantitation conducted on high-performance TLC plates for ‘staining, carbonisation, and densitometric analysis’ [106]. TLC, however, remains challenged by its low sensitivity and limited quantitative potential and throughput [107]. Further limitations are encountered in the identification and quantitation of individual TG(O) species as the entire TG(O) class tends to co-elute with the same R_f_ value.

Alternatively, saponification can be undertaken for the quantitation of total TG(O)s. In such cases, whole lipid fractions are hydrolysed via alkaline hydrolysis to release alkylglycerols into the hydrolysate following loss of the acyl chains. These mixtures can then be dried and reconstituted for mass spectrometry analysis, sometimes with a deuterated monoacylglycerol as the internal standard [62].

In a more recent report, TG(O)s in milk were identified from whole lipid extracts without additional extractions [63], an approach that might not be advisable for samples containing large amounts of interfering TGs. This interference has been observed in the study of other equally TG-rich matrices such as white adipose tissues (TG content of 98%-99%), resulting in the need for fractionation [108].

The challenge of removing sufficient TG interferences for the analysis of TG(O)s has been undertaken in tandem with the isolation of FAHFAs from human milk [55]. In the present study, the Folch method of extraction was used with acidification to obtain whole lipid extracts, followed by SPE on a silica cartridge. TG(O)s were eluted with 200:3 hexane: MTBE (v/v), and the remaining neutral lipids with 95:5 hexane: ethyl acetate (v/v). Although the recovery of TG(O)s were considerably low, the results were reproducible and TG(O) internal standards were added prior to extraction to help compensate for sample losses [55].

LC–MS^2^ analysis of alkylglycerol-type ether lipids can then be conducted with chromatographic separation on a reversed phase column and positive ionisation.

## Conclusions and future perspectives

With human milk as the optimal source of nutrition for infants, an accurate understanding of the human milk lipidome not limited to major lipid components could be important for unveiling the lipidomic perturbations underlying metabolic health and disease during infancy and later in life. While there are numerous classes of minor but bioactive lipids in human milk, we have focused on providing insight into two lipid classes exerting protective effects against diabetes, inflammation, and obesity: FAHFAs and TG(O)s. This review has covered the structural characteristics, biosynthesis and degradation, bioactivity, dietary sources, characterisations of FAHFAs and TG(O)s in human milk, and some of the methods used for the extraction of FAHFAs and TG(O)s.

Given that diabetes, inflammation, and obesity are deeply intertwined, establishing the profiles and roles of minor but bioactive lipids such as FAHFAs and TG(O)s could be of great interest for the prevention and management of metabolic dysfunction early in life. A concurrent analysis of both compounds in comprehensive cohorts could potentially help realise new associations between these lipid classes and reveal how perturbations in minor lipids are reflected in the health statuses of both mother and child. These studies could be conducted on human milk collected from nursing mothers with and without GDM, obesity, or pre-pregnancy diabetes. Longitudinal studies following the child from birth to the first few years of life could also be considered, with anthropometric measurements collected at regular intervals.

The method for the simultaneous extraction of FAHFAs and TG(O)s from the same sample has been validated and could potentially be modified to include the extraction of FAHFA-TGs, monoalkyl-monoacylglycerols, and other lipid classes that might be involved in the synthesis and storage of these compounds. With existing studies on human milk lipids being dominated by the more abundant lipid classes, analysis of minor lipids will better reflect the actual complexity of the human milk lipidome. Apart from establishing a starting point to probe into the roles of lipids in health and metabolism in early childhood, accurate characterisations will also bring us a step closer towards developing a gold standard for human milk fat substitutes.

## Perspectives

- Characterisations of lipids in human milk are largely focused on major lipids such as triglycerides, with much less work conducted on minor but bioactive lipid components. The overwhelming abundance of major lipid components could oversimplify the actual complexity of the human milk lipidome.
- FAHFAs and TG(O)s have emerged as novel lipid classes with protective effects against diabetes, inflammation, and childhood obesity. Studies on the bioactivity of FAHFAs and/or TG(O)s have been conducted in various matrices including lipid-rich adipose tissues and pancreatic β-cells. Characterisations of their profiles in human milk, however, are comparatively limited.
- The low concentration of FAHFAs and TG(O)s in human milk is one of the major challenges in characterising and establishing an accurate reference of the human milk lipidome. The development of reliable methods for the analysis of minor lipid fractions in human milk is thus crucial to further our understanding of these minor but bioactive lipids, and to identify possible dysregulations underlying health and disease.

## Supplementary Material

Supplementary Tables S1-S2

## Open Access

This article has been published open access under our Subscribe to Open programme, made possible through the support of our subscribing institutions, learn more here: https://portlandpress.com/pages/open_access_options_and_prices#conditional.

## Abbreviations

acyl-CoA: Acyl coenzyme A
ADTRP: Androgen-dependent tissue factor pathway inhibitor-regulating protein
AIG1: Androgen-induced gene 1
AMP: Adenosine monophosphate
AMPK: Adenosine monophosphate-activated protein kinase
ATGL: Adipose triglyceride lipase
ATM: Adipose tissue macrophage
CEL: Carboxyl ester lipase
CMEC: Cardiac microvascular endothelial cell
DGAT: Diglyceride acyltransferase
DG: Diacylglycerol
DMED: 2-dimethylaminoethylamine
ER: Endoplasmic reticulum
FA: Fatty acid
FAHFA: Fatty acid esters of hydroxy fatty acid
GDM: Gestational diabetes mellitus
GLP-1R: Glucagon-like peptide 1 receptor
GLUT4: Glucose transporter type 4
GPCR: G protein-coupled receptor
HFA: Hydroxy fatty acid
HSL: Hormone-sensitive lipase
IL-6: Interleukin-6
IP_3_: Inositol-1,4,5-triphosphate
LC–MS: Liquid chromatography–mass spectrometry
LKB1: Liver kinase B1
LPA: Lysophosphatidic acid
MAkG: Monoalkylglycerol
MAPK: Mitogen-activated protein kinase
Mdm2: Murine double minute 2
MFAT: Multifunctional O-acyltransferase
MGAT: Monoacylglycerol acyltransferase
MRM: Multiple reaction monitoring
MTBE: Methyl-tert-butyl-ether
OAHSA: Oleic acid ester of hydroxy stearic acid
PA: Phosphatidic acid
PAF: Platelet activating factor
PAFR: Platelet activating factor receptor
PAHSA: Palmitic acid ester of hydroxy stearic acid
PI3K: Phosphoinositide 3-kinase
PIP_2_: Phosphatidylinositol-4,5-biphosphate
PKD1: Protein kinase D1
PLC: Phospholipase C
PPARγ: Peroxisome proliferator-activated receptor gamma
Prdx6: Peroxiredoxin 6
SPE: Solid phase extraction
STAT3: Signal transducer and activator of transcription 3
STING: Stimulator of interferon genes
SWATH MS: Sequential window acquisition of all theoretical fragment ion spectra mass spectrometry
TFPI: Tissue factor pathway inhibitor
TG(O): Alkyl-diacylglycerol
TG: Triacylglycerol/triglyceride
TLC: Thin layer chromatography
Tyr: Tyrosine
ULK1: Unc-51-like kinase 1
