# Supplementary material for: Fatty acid esters of hydroxy fatty acids and alkyl-diacylglycerols: minor but bioactive components in human milk
Source: Biochem Soc Trans. 2026 Jun 8;54(6):699–714. doi: 10.1042/BST20260825 (PMC13259866; doi:10.1042/BST20260825)
Supplement: Supplementary Tables S1-S2 [file BST-2026-0825_supp.pdf]

Supplementary Table 1. FAHFAs reportedly present in human milk samples

FAHFA species/molecular species	Timepoint of sample collection	Mode of delivery	Concentration	Ref.
FAHFAs	1 month	*Unsp.*	1.01 µmol/L	(1)
FAHFA 2:0/17:2;O	Multiple timepoints	*Unsp.*	*Unsp.*	(2)
FAHFA 3:0/16:0;O	Multiple timepoints	*Unsp.*	*Unsp.*	(2)
FAHFA 4:0/17:0;O	Multiple timepoints	*Unsp.*	*Unsp.*	(2)
FAHFA 5:0/14:0;O	Multiple timepoints.	*Unsp.*	*Unsp.*	(2)
FAHFA 6:0/19:0;O	≤ 72 hours	*Unsp.*	*Unsp.*	(3)
FAHFA 7:0/12:0;O	≤ 72 hours	*Unsp.*	*Unsp.*	(3)
FAHFA 7:0/14:0;O	≤ 72 hours	*Unsp.*	*Unsp.*	(3)
FAHFA 10:0/16:0;O	Pooled 6 weeks, 3 months, 6 months	*Unsp.*	*Unsp.*	(4)
FAHFA 10:0/16:2;O	Multiple timepoints	*Unsp.*	*Unsp.*	(2)
FAHFA 10:0/18:0;O	Pooled 6 weeks, 3 months, 6 months	*Unsp.*	*Unsp.*	(4)
FAHFA 10:0/18:1;O	Multiple timepoints	*Unsp.*	*Unsp.*	(2)
FAHFA 12:0/16:0;O	Pooled 6 weeks, 3 months, 6 months	*Unsp.*	*Unsp.*	(4)
FAHFA 12:0/16:2;O	Multiple timepoints	*Unsp.*	*Unsp.*	(2)
FAHFA 12:0/18:0;O	Multiple timepoints	*Unsp.*	*Unsp.*	(2, 4)
FAHFA 12:0/18:1;O	Pooled 6 weeks, 3 months, 6 months	*Unsp.*	*Unsp.*	(4)
FAHFA 12:0/18:2;O	Multiple timepoints	*Unsp.*	*Unsp.*	(1, 2, 4)
FAHFA 14:0/14:0;O	Multiple timepoints	*Unsp.*	*Unsp.*	(5)

Values marked with ([1]) were estimated based on results presented in bar charts from (6) and line graphs from (7). Blank values marked with (*) were too low to estimate. Values reported either in terms of means or medians depending on publication. *Unsp.* = unspecified or non-specific

Supplementary Table 1 (continued). FAHFAs reportedly present in human milk samples

FAHFA species/molecular species	Timepoint of sample collection	Mode of delivery	Concentration	Ref.
FAHFA 14:0/1O(FA 14:0)	*Unsp.*	*Unsp.*	*Unsp.*	(5)
FAHFA 14:0/2O(FA 14:0)	*Unsp.*	*Unsp.*	*Unsp.*	(5)
FAHFA 14:0/9O(FA 14:0)	Colostrum	*Unsp.*	0.3 nmol/L	(5)
	Transitional milk	*Unsp.*	0.5 nmol/L	(5)
	Mature milk	*Unsp.*	0.5 nmol/L	(5)
FAHFA 14:0/16:0;O	Multiple timepoints	*Unsp.*	*Unsp.*	(4, 5)
FAHFA 14:0/1O(FA 16:0)	*Unsp.*	*Unsp.*	*Unsp.*	(5)
FAHFA 14:0/2O(FA 16:0)	*Unsp.*	*Unsp.*	*Unsp.*	(5)
FAHFA 14:0/3O(FA 16:0)	*Unsp.*	*Unsp.*	*Unsp.*	(5)
FAHFA 14:0/6O(FA 16:0)	*Unsp.*	*Unsp.*	*Unsp.*	(5)
FAHFA 14:0/7O(FA 16:0)	*Unsp.*	*Unsp.*	*Unsp.*	(5)
FAHFA 14:0/9O(FA 16:0)	Colostrum	*Unsp.*	2.2 nmol/L	(5)
	Transitional milk	*Unsp.*	4.0 nmol/L	(5)
	Mature milk	*Unsp.*	3.6 nmol/L	(5)
FAHFA 14:0/10O(FA 16:0)	Colostrum	*Unsp.*	0.3 nmol/L	(5)
	Transitional milk	*Unsp.*	0.9 nmol/L	(5)
	Mature milk	*Unsp.*	0.6 nmol/L	(5)
FAHFA 14:0/16:1;O	Multiple timepoints	*Unsp.*	*Unsp.*	(5)

Values marked with ([1]) were estimated based on results presented in bar charts from (6) and line graphs from (7). Blank values marked with (*) were too low to estimate. Values reported either in terms of means or medians depending on publication. *Unsp.* = unspecified or non-specific

Supplementary Table 1 (continued). FAHFAs reportedly present in human milk samples

FAHFA species/molecular species	Timepoint of sample collection	Mode of delivery	Concentration	Ref.
FAHFA 14:0/1O(FA 16:1)	*Unsp.*	*Unsp.*	*Unsp.*	(5)
FAHFA 14:0/2O(FA 16:1)	*Unsp.*	*Unsp.*	*Unsp.*	(5)
FAHFA 14:0/3O(FA 16:1)	*Unsp.*	*Unsp.*	*Unsp.*	(5)
FAHFA 14:0/18:0;O	Multiple timepoints	*Unsp.*	*Unsp.*	(4, 5)
FAHFA 14:0/1O(FA 18:0)	*Unsp.*	*Unsp.*	*Unsp.*	(5)
FAHFA 14:0/2O(FA 18:0)	*Unsp.*	*Unsp.*	*Unsp.*	(5)
FAHFA 14:0/9O(FA 18:0)	Colostrum	*Unsp.*	1.4 nmol/L	(5)
	Transitional milk	*Unsp.*	3.2 nmol/L	(5)
	Mature milk	*Unsp.*	2.9 nmol/L	(5)
FAHFA 14:0/18:1;O	Multiple timepoints	*Unsp.*	*Unsp.*	(4, 5)
FAHFA 14:0/1O(FA 18:1)	*Unsp.*	*Unsp.*	*Unsp.*	(5)
FAHFA 14:0/2O(FA 18:1)	*Unsp.*	*Unsp.*	*Unsp.*	(5)
FAHFA 14:0/3O(FA 18:1)	*Unsp.*	*Unsp.*	*Unsp.*	(5)
FAHFA 14:0/4O(FA 18:1)	*Unsp.*	*Unsp.*	*Unsp.*	(5)
FAHFA 14:0/5O(FA 18:1)	*Unsp.*	*Unsp.*	*Unsp.*	(5)
FAHFA 14:0/6O(FA 18:1)	*Unsp.*	*Unsp.*	*Unsp.*	(5)
FAHFA 14:0/18:2;O	Multiple timepoints	*Unsp.*	*Unsp.*	(4, 5)
FAHFA 14:0/1O(FA 18:2)	*Unsp.*	*Unsp.*	*Unsp.*	(5)

Values marked with ([1]) were estimated based on results presented in bar charts from (6) and line graphs from (7). Blank values marked with (*) were too low to estimate. Values reported either in terms of means or medians depending on publication. *Unsp.* = unspecified or non-specific

Supplementary Table 1 (continued). FAHFAs reportedly present in human milk samples

FAHFA species/molecular species	Timepoint of sample collection	Mode of delivery	Concentration	Ref.
FAHFA 14:0/2O(FA 18:2)	*Unsp.*	*Unsp.*	*Unsp.*	(5)
FAHFA 14:0/3O(FA 18:2)	*Unsp.*	*Unsp.*	*Unsp.*	(5)
FAHFA 14:0/4O(FA 18:2)	*Unsp.*	*Unsp.*	*Unsp.*	(5)
FAHFA 14:0/5O(FA 18:2)	*Unsp.*	*Unsp.*	*Unsp.*	(5)
FAHFA 14:0/24:0;O	Multiple timepoints	*Unsp.*	*Unsp.*	(2)
FAHFA 14:1/7:0;O	≤ 72 hours	*Unsp.*	*Unsp.*	(3)
FAHFA 14:1/16:0;O	Multiple timepoints	*Unsp.*	*Unsp.*	(5)
FAHFA 14:1/1O(FA 16:0)	*Unsp.*	*Unsp.*	*Unsp.*	(5)
FAHFA 14:1/2O(FA 16:0)	*Unsp.*	*Unsp.*	*Unsp.*	(5)
FAHFA 14:1/3O(FA 16:0)	*Unsp.*	*Unsp.*	*Unsp.*	(5)
FAHFA 14:1/4O(FA 16:0)	*Unsp.*	*Unsp.*	*Unsp.*	(5)
FAHFA 14:1/5O(FA 16:0)	*Unsp.*	*Unsp.*	*Unsp.*	(5)
FAHFA 14:1/6O(FA 16:0)	*Unsp.*	*Unsp.*	*Unsp.*	(5)
FAHFA 14:1/7O(FA 16:0)	*Unsp.*	*Unsp.*	*Unsp.*	(5)
FAHFA 14:1/18:0;O	Multiple timepoints	*Unsp.*	*Unsp.*	(5)
FAHFA 14:1/1O(FA 18:0)	*Unsp.*	*Unsp.*	*Unsp.*	(5)
FAHFA 14:1/2O(FA 18:0)	*Unsp.*	*Unsp.*	*Unsp.*	(5)
FAHFA 14:1/18:1;O	Multiple timepoints	*Unsp.*	*Unsp.*	(4, 5)

Values marked with ([1]) were estimated based on results presented in bar charts from (6) and line graphs from (7). Blank values marked with (*) were too low to estimate. Values reported either in terms of means or medians depending on publication. *Unsp.* = unspecified or non-specific

Supplementary Table 1 (continued). FAHFAs reportedly present in human milk samples

FAHFA species/molecular species	Timepoint of sample collection	Mode of delivery	Concentration	Ref.
FAHFA 14:1/1O(FA 18:1)	*Unsp.*	*Unsp.*	*Unsp.*	(5)
FAHFA 14:1/2O(FA 18:1)	*Unsp.*	*Unsp.*	*Unsp.*	(5)
FAHFA 14:1/3O(FA 18:1)	*Unsp.*	*Unsp.*	*Unsp.*	(5)
FAHFA 14:1/4O(FA 18:1)	*Unsp.*	*Unsp.*	*Unsp.*	(5)
FAHFA 15:0/5:0;O	Multiple timepoints	*Unsp.*	*Unsp.*	(2)
FAHFA 16:0/14:0;O	Multiple timepoints	*Unsp.*	*Unsp.*	(5)
FAHFA 16:0/1O(FA 14:0)	*Unsp.*	*Unsp.*	*Unsp.*	(5)
FAHFA 16:0/2O(FA 14:0)	*Unsp.*	*Unsp.*	*Unsp.*	(5)
FAHFA 16:0/9O(FA 14:0)	Colostrum	*Unsp.*	0.1 nmol/L	(5)
	Transitional milk	*Unsp.*	0.3 nmol/L	(5)
	Mature milk	*Unsp.*	0.2 nmol/L	(5)
FAHFA 16:0/16:0;O	Multiple timepoints	*Unsp.*	*Unsp.*	(5)
FAHFA 16:0/1O(FA 16:0)	*Unsp.*	*Unsp.*	*Unsp.*	(5)
FAHFA 16:0/2O(FA 16:0)	*Unsp.*	*Unsp.*	*Unsp.*	(5)
FAHFA 16:0/3O(FA 16:0)	*Unsp.*	*Unsp.*	*Unsp.*	(5)
FAHFA 16:0/4O(FA 16:0)	*Unsp.*	*Unsp.*	*Unsp.*	(5)
FAHFA 16:0/7O(FA 16:0)	*Unsp.*	*Unsp.*	*Unsp.*	(5)
FAHFA 16:0/8O(FA 16:0)	*Unsp.*	*Unsp.*	*Unsp.*	(5)

Values marked with ([1]) were estimated based on results presented in bar charts from (6) and line graphs from (7). Blank values marked with (*) were too low to estimate. Values reported either in terms of means or medians depending on publication. *Unsp.* = unspecified or non-specific

Supplementary Table 1 (continued). FAHFAs reportedly present in human milk samples

FAHFA species/molecular species	Timepoint of sample collection	Mode of delivery	Concentration	Ref.
FAHFA 16:0/9O(FA 16:0)	72 hours	Vaginal (38-41 weeks)	0.37 nmol/L[1]	(6)
	72 hours	Caesarean (38-41 weeks)	0.11 nmol/L[1]	(6)
	72 hours	Before 32 weeks	0.09 nmol/L[1]	(6)
	≤ 72 hours	*Unsp.*	18.0 nmol/L[1]<br>19.2 nmol/L[1] (BMI > 25)	(7)
	Colostrum	*Unsp.*	1.1 nmol/L	(5)
	Transitional milk	*Unsp.*	1.8 nmol/L	(5)
	Mature milk	*Unsp.*	1.3 nmol/L	(5)
	28 days	Vaginal (38-41 weeks)	0.27 nmol/L[1]	(6)
	28 days	Caesarean (38-41 weekseeks)	0.44 nmol/L[1]	(6)
	28 days	Before 32 weeks	0.34 nmol/L[1]	(6)
	42 days	*Unsp.*	11.3 nmol/L[1]<br>13.9 nmol/L[1] (BMI > 25)	(7)
	3 months	*Unsp.*	16.9 nmol/L[1]<br>15.2 nmol/L[1] (BMI > 25)	(7)
	36 weeks	Before 32 weeks	0.52 nmol/L[1]	(6)
FAHFA 16:0/10O(FA 16:0)	Colostrum	*Unsp.*	0.2 nmol/L	(5)
	Transitional milk	*Unsp.*	0.5 nmol/L	(5)
	Mature milk	*Unsp.*	0.4 nmol/L	(5)
FAHFA 16:0/16:1;O	Multiple timepoints	*Unsp.*	*Unsp.*	(4, 5)
FAHFA 16:0/1O(FA 16:1)	*Unsp.*	*Unsp.*	*Unsp.*	(5)

Values marked with ([1]) were estimated based on results presented in bar charts from (6) and line graphs from (7). Blank values marked with (*) were too low to estimate. Values reported either in terms of means or medians depending on publication. *Unsp.* = unspecified or non-specific

Supplementary Table 1 (continued). FAHFAs reportedly present in human milk samples

FAHFA species/molecular species	Timepoint of sample collection	Mode of delivery	Concentration	Ref.
FAHFA 16:0/2O(FA 16:1)	*Unsp.*	*Unsp.*	*Unsp.*	(5)
FAHFA 16:0/3O(FA 16:1)	*Unsp.*	*Unsp.*	*Unsp.*	(5)
FAHFA 16:0/4O(FA 16:1)	*Unsp.*	*Unsp.*	*Unsp.*	(5)
FAHFA 16:0/16:2;O	Multiple timepoints	*Unsp.*	*Unsp.*	(5)
FAHFA 16:0/1O(FA 16:2)	*Unsp.*	*Unsp.*	*Unsp.*	(5)
FAHFA 16:0/18:0;O	Multiple timepoints	*Unsp.*	*Unsp.*	(2, 4, 5)
FAHFA 16:0/1O(FA 18:0)	*Unsp.*	*Unsp.*	*Unsp.*	(5)
FAHFA 16:0/4O(FA 18:0)	*Unsp.*	*Unsp.*	*Unsp.*	(5)
FAHFA 16:0/5O(FA 18:0)	≤ 72 hours	*Unsp.*	*Unsp.*	(3)
	72 hours	Vaginal (38-41 weeks)	13.7 nmol/L[1]	(6)
	72 hours	Caesarean (38-41 weeks)	2.2 nmol/L[1]	(6)
	72 hours	Before 32 weeks	0.8 nmol/L[1]	(6)
	≤ 72 hours	*Unsp.*	13.2 nmol/L[1]<br>20.4 nmol/L[1] (BMI > 25)	(7)
	28 days	Vaginal (38-41 weeks)	4.7 nmol/L[1]	(6)
	28 days	Caesarean (38-41 weeks)	3.5 nmol/L[1]	(6)
	28 days	Before 32 weeks	1.6 nmol/L[1]	(6)
	42 days	*Unsp.*	7.3 nmol/L[1]<br>6.7 nmol/L[1] (BMI > 25)	(7)
	3 months	*Unsp.*	3.2 nmol/L[1]<br>5 nmol/L* (BMI > 25)	(7)
	36 weeks	Before 32 weeks	2.4 nmol/L[1]	(6)

Values marked with ([1]) were estimated based on results presented in bar charts from (6) and line graphs from (7). Blank values marked with (*) were too low to estimate. Values reported either in terms of means or medians depending on publication. *Unsp.* = unspecified or non-specific

Supplementary Table 1 (continued). FAHFAs reportedly present in human milk samples

FAHFA species/molecular species	Timepoint of sample collection	Mode of delivery	Concentration	Ref.
FAHFA 16:0/9O(FA 18:0)	72 hours	Vaginal (38-41 weeks)	3.2 nmol/L[1]	(6)
	72 hours	Caesarean (38-41 weeks)	2.5 nmol/L[1]	(6)
	72 hours	Before 32 weeks	1.3 nmol/L[1]	(6)
	≤ 72 hours	*Unsp.*	3.7 nmol/L[1]<br>3.1 nmol/L[1] (BMI > 25)	(7)
	Colostrum	*Unsp.*	0.8 nmol/L	(5)
	Transitional milk	*Unsp.*	1.3 nmol/L	(5)
	Mature milk	*Unsp.*	1.1 nmol/L	(5)
	28 days	Vaginal (38-41 weeks)	3.0 nmol/L[1]	(6)
	28 days	Caesarean (38-41 weeks)	4.5 nmol/L[1]	(6)
	28 days	Before 32 weeks	2.3 nmol/L[1]	(6)
	42 days	*Unsp.*	3.6 nmol/L[1]<br>3.9 nmol/L[1] (BMI > 25)	(7)
	3 months	*Unsp.*	3.2 nmol/L[1]<br>2.7 nmol/L[1] (BMI > 25)	(7)
	36 weeks	Before 32 weeks	1.6 nmol/L[1]	(6)

Values marked with ([1]) were estimated based on results presented in bar charts from (6) and line graphs from (7). Blank values marked with (*) were too low to estimate. Values reported either in terms of means or medians depending on publication. *Unsp.* = unspecified or non-specific

Supplementary Table 1 (continued). FAHFAs reportedly present in human milk samples

FAHFA species/molecular species	Timepoint of sample collection	Mode of delivery	Concentration	Ref.
FAHFA 16:0/10O(FA 18:0)	72 hours	Vaginal (38-41 weeks)	4.4 nmol/L[1]	(6)
	72 hours	Caesarean (38-41 weeks)	3.2 nmol/L[1]	(6)
	72 hours	Before 32 weeks	4.4 nmol/L[1]	(6)
	Colostrum	*Unsp.*	1.0 nmol/L	(5)
	Transitional milk	*Unsp.*	1.2 nmol/L	(5)
	Mature milk	*Unsp.*	1.5 nmol/L	(5)
	28 days	Vaginal (38-41 weeks)	4.8 nmol/L[1]	(6)
	28 days	Caesarean (38-41 weeks)	8.3 nmol/L[1]	(6)
	28 days	Before 32 weeks	9.2 nmol/L[1]	(6)
	36 weeks	Before 32 weeks	5.1 nmol/L[1]	(6)
FAHFA 16:0/12O(FA 18:0)	Colostrum	*Unsp.*	0.3 nmol/L	(5)
	Transitional milk	*Unsp.*	0.4 nmol/L	(5)
	Mature milk	*Unsp.*	0.2 nmol/L	(5)
FAHFA 16:0/13O(FA 18:0)	Colostrum	*Unsp.*	0.2 nmol/L	(5)
	Transitional milk	*Unsp.*	0.2 nmol/L	(5)
	Mature milk	*Unsp.*	0.1 nmol/L	(5)
FAHFA 16:0/18:1;O	Multiple timepoints	*Unsp.*	*Unsp.*	(4, 5)
FAHFA 16:0/1O(FA 18:1)	*Unsp.*	*Unsp.*	*Unsp.*	(5)

Values marked with ([1]) were estimated based on results presented in bar charts from (6) and line graphs from (7). Blank values marked with (*) were too low to estimate. Values reported either in terms of means or medians depending on publication. *Unsp.* = unspecified or non-specific

Supplementary Table 1 (continued). FAHFAs reportedly present in human milk samples

FAHFA species/molecular species	Timepoint of sample collection	Mode of delivery	Concentration	Ref.
FAHFA 16:0/2O(FA 18:1)	*Unsp.*	*Unsp.*	*Unsp.*	(5)
FAHFA 16:0/3O(FA 18:1)	*Unsp.*	*Unsp.*	*Unsp.*	(5)
FAHFA 16:0/4O(FA 18:1)	*Unsp.*	*Unsp.*	*Unsp.*	(5)
FAHFA 16:0/5O(FA 18:1)	*Unsp.*	*Unsp.*	*Unsp.*	(5)
FAHFA 16:0/6O(FA 18:1)	*Unsp.*	*Unsp.*	*Unsp.*	(5)
FAHFA 16:0/7O(FA 18:1)	*Unsp.*	*Unsp.*	*Unsp.*	(5)
FAHFA 16:0/18:2;O	Multiple timepoints	*Unsp.*	*Unsp.*	(2, 3, 5)
FAHFA 16:0/1O(FA 18:2)	*Unsp.*	*Unsp.*	*Unsp.*	(5)
FAHFA 16:0/2O(FA 18:2)	*Unsp.*	*Unsp.*	*Unsp.*	(5)
FAHFA 16:0/3O(FA 18:2)	*Unsp.*	*Unsp.*	*Unsp.*	(5)
FAHFA 16:0/4O(FA 18:2)	*Unsp.*	*Unsp.*	*Unsp.*	(5)
FAHFA 16:0/5O(FA 18:2)	*Unsp.*	*Unsp.*	*Unsp.*	(5)
FAHFA 16:0/6O(FA 18:2)	*Unsp.*	*Unsp.*	*Unsp.*	(5)
FAHFA 16:0/7O(FA 18:2)	*Unsp.*	*Unsp.*	*Unsp.*	(5)
FAHFA 16:0/22:4;O	Multiple timepoints	*Unsp.*	*Unsp.*	(2)
FAHFA 16:0/24:4;O	Multiple timepoints	*Unsp.*	*Unsp.*	(2)
FAHFA 16:1/14:0;O	Multiple timepoints	*Unsp.*	*Unsp.*	(5)
FAHFA 16:1/1O(FA 14:0)	*Unsp.*	*Unsp.*	*Unsp.*	(5)

Values marked with ([1]) were estimated based on results presented in bar charts from (6) and line graphs from (7). Blank values marked with (*) were too low to estimate. Values reported either in terms of means or medians depending on publication. *Unsp.* = unspecified or non-specific

Supplementary Table 1 (continued). FAHFAs reportedly present in human milk samples

FAHFA species/molecular species	Timepoint of sample collection	Mode of delivery	Concentration	Ref.
FAHFA 16:1/2O(FA 14:0)	*Unsp.*	*Unsp.*	*Unsp.*	(5)
FAHFA 16:1/3O(FA 14:0)	*Unsp.*	*Unsp.*	*Unsp.*	(5)
FAHFA 16:1/4O(FA 14:0)	*Unsp.*	*Unsp.*	*Unsp.*	(5)
FAHFA 16:1/5O(FA 14:0)	*Unsp.*	*Unsp.*	*Unsp.*	(5)
FAHFA 16:1/16:0;O	Multiple timepoints	*Unsp.*	*Unsp.*	(2, 4, 5)
FAHFA 16:1/1O(FA 16:0)	*Unsp.*	*Unsp.*	*Unsp.*	(5)
FAHFA 16:1/2O(FA 16:0)	*Unsp.*	*Unsp.*	*Unsp.*	(5)
FAHFA 16:1/3O(FA 16:0)	*Unsp.*	*Unsp.*	*Unsp.*	(5)
FAHFA 16:1/4O(FA 16:0)	*Unsp.*	*Unsp.*	*Unsp.*	(5)
FAHFA 16:1/5O(FA 16:0)	*Unsp.*	*Unsp.*	*Unsp.*	(5)
FAHFA 16:1/6O(FA 16:0)	*Unsp.*	*Unsp.*	*Unsp.*	(5)
FAHFA 16:1/7O(FA 16:0)	*Unsp.*	*Unsp.*	*Unsp.*	(5)
FAHFA 16:1/16:1;O	Multiple timepoints	*Unsp.*	*Unsp.*	(2, 4, 5)
FAHFA 16:1/1O(FA 16:1)	*Unsp.*	*Unsp.*	*Unsp.*	(5)
FAHFA 16:1/2O(FA 16:1)	*Unsp.*	*Unsp.*	*Unsp.*	(5)
FAHFA 16:1/3O(FA 16:1)	*Unsp.*	*Unsp.*	*Unsp.*	(5)
FAHFA 16:1/4O(FA 16:1)	*Unsp.*	*Unsp.*	*Unsp.*	(5)
FAHFA 16:1/5O(FA 16:1)	*Unsp.*	*Unsp.*	*Unsp.*	(5)

Values marked with ([1]) were estimated based on results presented in bar charts from (6) and line graphs from (7). Blank values marked with (*) were too low to estimate. Values reported either in terms of means or medians depending on publication. *Unsp.* = unspecified or non-specific

Supplementary Table 1 (continued). FAHFAs reportedly present in human milk samples

FAHFA species/molecular species	Timepoint of sample collection	Mode of delivery	Concentration	Ref.
FAHFA 16:1/6O(FA 16:1)	*Unsp.*	*Unsp.*	*Unsp.*	(5)
FAHFA 16:1/16:2;O	Multiple timepoints	*Unsp.*	*Unsp.*	(5)
FAHFA 16:1/1O(FA 16:2)	*Unsp.*	*Unsp.*	*Unsp.*	(5)
FAHFA 16:1/2O(FA 16:2)	*Unsp.*	*Unsp.*	*Unsp.*	(5)
FAHFA 16:1/18:0;O	Multiple timepoints	*Unsp.*	*Unsp.*	(4, 5)
FAHFA 16:1/1O(FA 18:0)	*Unsp.*	*Unsp.*	*Unsp.*	(5)
FAHFA 16:1/9O(FA 18:0)	≤ 72 hours	*Unsp.*	2.0 nmol/L[1]<br>1.8 nmol/L[1] (BMI > 25)	(7)
	Colostrum	*Unsp.*	2.2 nmol/L	(5)
	Transitional milk	*Unsp.*	5.9 nmol/L	(5)
	Mature milk	*Unsp.*	4.4 nmol/L	(5)
	42 days	*Unsp.*	12.4 nmol/L[1]<br>12.4 nmol/L[1] (BMI > 25)	(7)
	3 months	*Unsp.*	9.4 nmol/L[1]<br>8.1 nmol/L[1] (BMI > 25)	(7)
FAHFA 16:1/10O(FA 18:0)	Colostrum	*Unsp.*	2.1 nmol/L	(5)
	Transitional milk	*Unsp.*	4.2 nmol/L	(5)
	Mature milk	*Unsp.*	4.4 nmol/L	(5)
FAHFA 16:1/18:1;O	Multiple timepoints	*Unsp.*	*Unsp.*	(4, 5)
FAHFA 16:1/1O(FA 18:1)	*Unsp.*	*Unsp.*	*Unsp.*	(5)
FAHFA 16:1/2O(FA 18:1)	*Unsp.*	*Unsp.*	*Unsp.*	(5)

Values marked with ([1]) were estimated based on results presented in bar charts from (6) and line graphs from (7). Blank values marked with (*) were too low to estimate. Values reported either in terms of means or medians depending on publication. *Unsp.* = unspecified or non-specific

Supplementary Table 1 (continued). FAHFAs reportedly present in human milk samples

FAHFA species/molecular species	Timepoint of sample collection	Mode of delivery	Concentration	Ref.
FAHFA 16:1/3O(FA 18:1)	*Unsp.*	*Unsp.*	*Unsp.*	(5)
FAHFA 16:1/4O(FA 18:1)	*Unsp.*	*Unsp.*	*Unsp.*	(5)
FAHFA 16:1/5O(FA 18:1)	*Unsp.*	*Unsp.*	*Unsp.*	(5)
FAHFA 16:1/6O(FA 18:1)	*Unsp.*	*Unsp.*	*Unsp.*	(5)
FAHFA 16:1/18:2;O	Multiple timepoints	*Unsp.*	*Unsp.*	(4, 5)
FAHFA 16:1/1O(FA 18:2)	*Unsp.*	*Unsp.*	*Unsp.*	(5)
FAHFA 16:1/2O(FA 18:2)	*Unsp.*	*Unsp.*	*Unsp.*	(5)
FAHFA 16:1/3O(FA 18:2)	*Unsp.*	*Unsp.*	*Unsp.*	(5)
FAHFA 16:1/4O(FA 18:2)	*Unsp.*	*Unsp.*	*Unsp.*	(5)
FAHFA 16:1/5O(FA 18:2)	*Unsp.*	*Unsp.*	*Unsp.*	(5)
FAHFA 16:1/6O(FA 18:2)	*Unsp.*	*Unsp.*	*Unsp.*	(5)
FAHFA 16:1/22:4;O	Multiple timepoints	*Unsp.*	*Unsp.*	(2)
FAHFA 16:2/21:2;O	≤ 72 hours	*Unsp.*	*Unsp.*	(3)
FAHFA 16:2/26:4;O	Multiple timepoints	*Unsp.*	*Unsp.*	(2)
FAHFA 17:1/16:0;O	Multiple timepoints	*Unsp.*	*Unsp.*	(5)
FAHFA 17:1/1O(FA 16:0)	*Unsp.*	*Unsp.*	*Unsp.*	(5)
FAHFA 17:1/2O(FA 16:0)	*Unsp.*	*Unsp.*	*Unsp.*	(5)
FAHFA 17:1/18:0;O	Multiple timepoints	*Unsp.*	*Unsp.*	(5)

Values marked with ([1]) were estimated based on results presented in bar charts from (6) and line graphs from (7). Blank values marked with (*) were too low to estimate. Values reported either in terms of means or medians depending on publication. *Unsp.* = unspecified or non-specific

Supplementary Table 1 (continued). FAHFAs reportedly present in human milk samples

FAHFA species/molecular species	Timepoint of sample collection	Mode of delivery	Concentration	Ref.
FAHFA 17:1/1O(FA 18:0)	*Unsp.*	*Unsp.*	*Unsp.*	(5)
FAHFA 17:1/2O(FA 18:0)	*Unsp.*	*Unsp.*	*Unsp.*	(5)
FAHFA 18:0/16:0;O	Multiple timepoints	*Unsp.*	*Unsp.*	(4, 5)
FAHFA 18:0/1O(FA 16:0)	*Unsp.*	*Unsp.*	*Unsp.*	(5)
FAHFA 18:0/2O(FA 16:0)	*Unsp.*	*Unsp.*	*Unsp.*	(5)
FAHFA 18:0/3O(FA 16:0)	*Unsp.*	*Unsp.*	*Unsp.*	(5)
FAHFA 18:0/5O(FA 16:0)	*Unsp.*	*Unsp.*	*Unsp.*	(5)
FAHFA 18:0/6O(FA 16:0)	*Unsp.*	*Unsp.*	*Unsp.*	(5)
FAHFA 18:0/9O(FA 16:0)	Colostrum	*Unsp.*	0.4 nmol/L	(5)
	Transitional milk	*Unsp.*	0.6 nmol/L	(5)
	Mature milk	*Unsp.*	0.6 nmol/L	(5)
FAHFA 18:0/16:1;O	1 month	*Unsp.*	0.54µmol/L	(1)
FAHFA 18:0/18:0;O	Multiple timepoints	*Unsp.*	*Unsp.*	(5)
FAHFA 18:0/5O(FA 18:0)	≤ 72 hours	*Unsp.*	*Unsp.*	(3)
FAHFA 18:0/9O(FA 18:0)	≤ 72 hours	*Unsp.*	1.6 nmol/L[1]<br>2.9 nmol/L[1] (BMI > 25)	(7)
	42 days	*Unsp.*	4.9 nmol/L[1]<br>8.0 nmol/L[1] (BMI > 25)	(7)
	3 months	*Unsp.*	15.4 nmol/L[1]<br>14 nmol/L[1] (BMI > 25)	(7)
	Unsp.	*Unsp.*	*Unsp.*	(5)

Values marked with ([1]) were estimated based on results presented in bar charts from (6) and line graphs from (7). Blank values marked with (*) were too low to estimate. Values reported either in terms of means or medians depending on publication. *Unsp.* = unspecified or non-specific

Supplementary Table 1 (continued). FAHFAs reportedly present in human milk samples

FAHFA species/molecular species	Timepoint of sample collection	Mode of delivery	Concentration	Ref.
FAHFA 18:0/10O(FA 18:0)	*Unsp.*	*Unsp.*	*Unsp.*	(5)
FAHFA 18:0/18:1;O	Multiple timepoints	*Unsp.*	0.22 µmol/L at 1 month	(1, 5)
FAHFA 18:0/1O(FA 18:1)	*Unsp.*	*Unsp.*	*Unsp.*	(5)
FAHFA 18:0/2O(FA 18:1)	*Unsp.*	*Unsp.*	*Unsp.*	(5)
FAHFA 18:0/18:2;O	Multiple timepoints	*Unsp.*	*Unsp.*	(5)
FAHFA 18:0/1O(FA 18:2)	*Unsp.*	*Unsp.*	*Unsp.*	(5)
FAHFA 18:0/2O(FA 18:2)	*Unsp.*	*Unsp.*	*Unsp.*	(5)
FAHFA 18:0/3O(FA 18:2)	*Unsp.*	*Unsp.*	*Unsp.*	(5)
FAHFA 18:0/20:2;O	Multiple timepoints	*Unsp.*	*Unsp.*	(2)
FAHFA 18:1/8:0;O	Multiple timepoints	*Unsp.*	*Unsp.*	(2)
FAHFA 18:1/12:0;O	Multiple timepoints	*Unsp.*	*Unsp.*	(5)
FAHFA 18:1/1O(FA 12:0)	*Unsp.*	*Unsp.*	*Unsp.*	(5)
FAHFA 18:1/2O(FA 12:0)	*Unsp.*	*Unsp.*	*Unsp.*	(5)
FAHFA 18:1/3O(FA 12:0)	*Unsp.*	*Unsp.*	*Unsp.*	(5)
FAHFA 18:1/4O(FA 12:0)	*Unsp.*	*Unsp.*	*Unsp.*	(5)
FAHFA 18:1/5O(FA 12:0)	*Unsp.*	*Unsp.*	*Unsp.*	(5)
FAHFA 18:1/6O(FA 12:0)	*Unsp.*	*Unsp.*	*Unsp.*	(5)
FAHFA 18:1/7O(FA 12:0)	*Unsp.*	*Unsp.*	*Unsp.*	(5)

Values marked with ([1]) were estimated based on results presented in bar charts from (6) and line graphs from (7). Blank values marked with (*) were too low to estimate. Values reported either in terms of means or medians depending on publication. *Unsp.* = unspecified or non-specific

Supplementary Table 1 (continued). FAHFAs reportedly present in human milk samples

FAHFA species/molecular species	Timepoint of sample collection	Mode of delivery	Concentration	Ref.
FAHFA 18:1/14:0;O	Multiple timepoints	*Unsp.*	*Unsp.*	(2, 5)
FAHFA 18:1/1O(FA 14:0)	*Unsp.*	*Unsp.*	*Unsp.*	(5)
FAHFA 18:1/2O(FA 14:0)	*Unsp.*	*Unsp.*	*Unsp.*	(5)
FAHFA 18:1/3O(FA 14:0)	*Unsp.*	*Unsp.*	*Unsp.*	(5)
FAHFA 18:1/4O(FA 14:0)	*Unsp.*	*Unsp.*	*Unsp.*	(5)
FAHFA 18:1/5O(FA 14:0)	*Unsp.*	*Unsp.*	*Unsp.*	(5)
FAHFA 18:1/6O(FA 14:0)	*Unsp.*	*Unsp.*	*Unsp.*	(5)
FAHFA 18:1/7O(FA 14:0)	*Unsp.*	*Unsp.*	*Unsp.*	(5)
FAHFA 18:1/8O(FA 14:0)	*Unsp.*	*Unsp.*	*Unsp.*	(5)
FAHFA 18:1/14:1;O	Multiple timepoints	*Unsp.*	*Unsp.*	(2, 4, 5)
FAHFA 18:1/1O(FA 14:1)	*Unsp.*	*Unsp.*	*Unsp.*	(5)
FAHFA 18:1/2O(FA 14:1)	*Unsp.*	*Unsp.*	*Unsp.*	(5)
FAHFA 18:1/3O(FA 14:1)	*Unsp.*	*Unsp.*	*Unsp.*	(5)
FAHFA 18:1/4O(FA 14:1)	*Unsp.*	*Unsp.*	*Unsp.*	(5)
FAHFA 18:1/5O(FA 14:1)	*Unsp.*	*Unsp.*	*Unsp.*	(5)
FAHFA 18:1/6O(FA 14:1)	*Unsp.*	*Unsp.*	*Unsp.*	(5)
FAHFA 18:1/7O(FA 14:1)	*Unsp.*	*Unsp.*	*Unsp.*	(5)
FAHFA 18:1/14:2;O	Multiple timepoints	*Unsp.*	*Unsp.*	(5)

Values marked with ([1]) were estimated based on results presented in bar charts from (6) and line graphs from (7). Blank values marked with (*) were too low to estimate. Values reported either in terms of means or medians depending on publication. *Unsp.* = unspecified or non-specific

Supplementary Table 1 (continued). FAHFAs reportedly present in human milk samples

FAHFA species/molecular species	Timepoint of sample collection	Mode of delivery	Concentration	Ref.
FAHFA 18:1/1O(FA 14:2)	*Unsp.*	*Unsp.*	*Unsp.*	(5)
FAHFA 18:1/2O(FA 14:2)	*Unsp.*	*Unsp.*	*Unsp.*	(5)
FAHFA 18:1/15:0;O	Multiple timepoints	*Unsp.*	*Unsp.*	(5)
FAHFA 18:1/1O(FA 15:0)	*Unsp.*	*Unsp.*	*Unsp.*	(5)
FAHFA 18:1/2O(FA 15:0)	*Unsp.*	*Unsp.*	*Unsp.*	(5)
FAHFA 18:1/3O(FA 15:0)	*Unsp.*	*Unsp.*	*Unsp.*	(5)
FAHFA 18:1/4O(FA 15:0)	*Unsp.*	*Unsp.*	*Unsp.*	(5)
FAHFA 18:1/16:0;O	Multiple timepoints	*Unsp.*	*Unsp.*	(2, 4, 5)
FAHFA 18:1/1O(FA 16:0)	*Unsp.*	*Unsp.*	*Unsp.*	(5)
FAHFA 18:1/2O(FA 16:0)	*Unsp.*	*Unsp.*	*Unsp.*	(5)
FAHFA 18:1/3O(FA 16:0)	*Unsp.*	*Unsp.*	*Unsp.*	(5)
FAHFA 18:1/4O(FA 16:0)	*Unsp.*	*Unsp.*	*Unsp.*	(5)
FAHFA 18:1/5O(FA 16:0)	*Unsp.*	*Unsp.*	*Unsp.*	(5)
FAHFA 18:1/6O(FA 16:0)	*Unsp.*	*Unsp.*	*Unsp.*	(5)
FAHFA 18:1/7O(FA 16:0)	*Unsp.*	*Unsp.*	*Unsp.*	(5)
FAHFA 18:1/9O(FA 16:0)	≤ 72 hours	*Unsp.*	2.3 nmol/L[1]<br>2.1 nmol/L[1] (BMI > 25)	(7)
	42 days	*Unsp.*	1.3 nmol/L[1]<br>1.2 nmol/L[1] (BMI > 25)	(7)
	3 months	*Unsp.*	1.3 nmol/L[1]<br>1 nmol/L[1] (BMI > 25)	(7)

Values marked with ([1]) were estimated based on results presented in bar charts from (6) and line graphs from (7). Blank values marked with (*) were too low to estimate. Values reported either in terms of means or medians depending on publication. *Unsp.* = unspecified or non-specific

Supplementary Table 1 (continued). FAHFAs reportedly present in human milk samples

FAHFA species/molecular species	Timepoint of sample collection	Mode of delivery	Concentration	Ref.
FAHFA 18:1/16:1;O	Multiple timepoints	*Unsp.*	*Unsp.*	(4, 5)
FAHFA 18:1/1O(FA 16:1)	*Unsp.*	*Unsp.*	*Unsp.*	(5)
FAHFA 18:1/2O(FA 16:1)	*Unsp.*	*Unsp.*	*Unsp.*	(5)
FAHFA 18:1/3O(FA 16:1)	*Unsp.*	*Unsp.*	*Unsp.*	(5)
FAHFA 18:1/4O(FA 16:1)	*Unsp.*	*Unsp.*	*Unsp.*	(5)
FAHFA 18:1/5O(FA 16:1)	*Unsp.*	*Unsp.*	*Unsp.*	(5)
FAHFA 18:1/6O(FA 16:1)	*Unsp.*	*Unsp.*	*Unsp.*	(5)
FAHFA 18:1/7O(FA 16:1)	*Unsp.*	*Unsp.*	*Unsp.*	(5)
FAHFA 18:1/8O(FA 16:1)	*Unsp.*	*Unsp.*	*Unsp.*	(5)
FAHFA 18:1/9O(FA 16:1)	*Unsp.*	*Unsp.*	*Unsp.*	(5)
FAHFA 18:1/16:2;O	Multiple timepoints	*Unsp.*	*Unsp.*	(2, 4, 5)
FAHFA 18:1/1O(FA 16:2)	*Unsp.*	*Unsp.*	*Unsp.*	(5)
FAHFA 18:1/2O(FA 16:2)	*Unsp.*	*Unsp.*	*Unsp.*	(5)
FAHFA 18:1/3O(FA 16:2)	*Unsp.*	*Unsp.*	*Unsp.*	(5)
FAHFA 18:1/4O(FA 16:2)	*Unsp.*	*Unsp.*	*Unsp.*	(5)
FAHFA 18:1/5O(FA 16:2)	*Unsp.*	*Unsp.*	*Unsp.*	(5)
FAHFA 18:1/17:0;O	Multiple timepoints	*Unsp.*	*Unsp.*	(5)
FAHFA 18:1/1O(FA 17:0)	*Unsp.*	*Unsp.*	*Unsp.*	(5)

Values marked with ([1]) were estimated based on results presented in bar charts from (6) and line graphs from (7). Blank values marked with (*) were too low to estimate. Values reported either in terms of means or medians depending on publication. *Unsp.* = unspecified or non-specific

Supplementary Table 1 (continued). FAHFAs reportedly present in human milk samples

FAHFA species/molecular species	Timepoint of sample collection	Mode of delivery	Concentration	Ref.
FAHFA 18:1/18:0;O	Multiple timepoints	*Unsp.*	*Unsp.*	(3-5)
FAHFA 18:1/1O(FA 18:0)	*Unsp.*	*Unsp.*	*Unsp.*	(5)
FAHFA 18:1/2O(FA 18:0)	*Unsp.*	*Unsp.*	*Unsp.*	(5)
FAHFA 18:1/5O(FA 18:0)	*Unsp.*	*Unsp.*	*Unsp.*	(5)
FAHFA 18:1/8O(FA 18:0)	*Unsp.*	*Unsp.*	*Unsp.*	(5)
FAHFA 18:1/9O(FA 18:0)	72 hours	Vaginal (38-41 weeks)	1.2 nmol/L[1]	(6)
	72 hours	Caesarean (38-41 weeks)	*	(6)
	72 hours	Before 32 weeks	*	(6)
	≤ 72 hours	*Unsp.*	3.5 nmol/L[1]<br>2.9 nmol/L[1] (BMI > 25)	(7)
	Colostrum	*Unsp.*	21.4 nmol/L	(5)
	Transitional milk	*Unsp.*	51.1 nmol/L	(5)
	Mature milk	*Unsp.*	46.6 nmol/L	(5)
	28 days	Vaginal (38-41 weeks)	7.9 nmol/L[1]	(6)
	28 days	Caesarean (38-41 weeks)	18.3 nmol/L[1]	(6)
	28 days	Before 32 weeks	16.6 nmol/L[1]	(6)
	42 days	*Unsp.*	10.9 nmol/L[1]<br>10.4 nmol/L[1] (BMI > 25)	(7)
	3 months	*Unsp.*	12.2 nmol/L[1]<br>11.8 nmol/L[1] (BMI > 25)	(7)
	36 weeks	Before 32 weeks	13.5 nmol/L[1]	(6)

Values marked with ([1]) were estimated based on results presented in bar charts from (6) and line graphs from (7). Blank values marked with (*) were too low to estimate. Values reported either in terms of means or medians depending on publication. *Unsp.* = unspecified or non-specific

Supplementary Table 1 (continued). FAHFAs reportedly present in human milk samples

FAHFA species/molecular species	Timepoint of sample collection	Mode of delivery	Concentration	Ref.
FAHFA 18:1/10O(FA 18:0)	72 hours	Vaginal (38-**41 weeks**)	1.9 nmol/L[1]	(6)
	72 hours	Caesarean (38-41 weeks)	*	(6)
	72 hours	Before 32 weeks	1.6 nmol/L[1]	(6)
	≤ 72 hours	*Unsp.*	2.2 nmol/L[1]<br>1.7 nmol/L[1] (BMI > 25)	(7)
	Colostrum	*Unsp.*	21.5 nmol/L	(5)
	Transitional milk	*Unsp.*	30.2 nmol/L	(5)
	Mature milk	*Unsp.*	39.9 nmol/L	(5)
	28 days	Vaginal (38-41 weeks)	8.9 nmol/L[1]	(6)
	28 days	Caesarean (38-41 weeks)	21.1 nmol/L[1]	(6)
	28 days	Before 32 weeks	26.4 nmol/L[1]	(6)
	42 days	*Unsp.*	8.8 nmol/L[1]<br>7.9 nmol/L[1] (BMI > 25)	(7)
	3 months	*Unsp.*	10.1 nmol/L[1]<br>9.9 nmol/L[1] (BMI > 25)	(7)
	36 weeks	Before 32 weeks	10.5 nmol/L[1]	(6)

Values marked with ([1]) were estimated based on results presented in bar charts from (6) and line graphs from (7). Blank values marked with (*) were too low to estimate. Values reported either in terms of means or medians depending on publication. *Unsp.* = unspecified or non-specific

Supplementary Table 1 (continued). FAHFAs reportedly present in human milk samples

FAHFA species/molecular species	Timepoint of sample collection	Mode of delivery	Concentration	Ref.
FAHFA 18:1/12O(FA 18:0) OR FAHFA 18:1/13O(FA 18:0)	72 hours	Vaginal (38-41 weeks)	4.0 nmol/L[1]	(6)
	72 hours	Caesarean (38-41 weeks)	1.1 nmol/L[1]	(6)
	72 hours	Before 32 weeks	1.0 nmol/L[1]	(6)
	28 days	Vaginal (38-41 weeks)	7.6 nmol/L[1]	(6)
	28 days	Caesarean (38-41 weeks)	12.8 nmol/L[1]	(6)
	28 days	Before 32 weeks	12.2 nmol/L[1]	(6)
	36 weeks	Before 32 weeks	9.4 nmol/L[1]	(6)
FAHFA 18:1/12O(FA 18:0)	Colostrum	*Unsp.*	2.3 nmol/L	(5)
	Transitional milk	*Unsp.*	3.8 nmol/L	(5)
	Mature milk	*Unsp.*	4.0 nmol/L	(5)
FAHFA 18:1/13O(FA 18:0)	Colostrum	*Unsp.*	2.6 nmol/L	(5)
	Transitional milk	*Unsp.*	5.5 nmol/L	(5)
	Mature milk	*Unsp.*	4.8 nmol/L	(5)
FAHFA 18:1/18:1;O	Multiple timepoints	*Unsp.*	*Unsp.*	(2, 4, 5)
FAHFA 18:1/1O(FA 18:1)	*Unsp.*	*Unsp.*	*Unsp.*	(5)
FAHFA 18:1/2O(FA 18:1)	*Unsp.*	*Unsp.*	*Unsp.*	(5)
FAHFA 18:1/3O(FA 18:1)	*Unsp.*	*Unsp.*	*Unsp.*	(5)
FAHFA 18:1/4O(FA 18:1)	*Unsp.*	*Unsp.*	*Unsp.*	(5)

Values marked with ([1]) were estimated based on results presented in bar charts from (6) and line graphs from (7). Blank values marked with (*) were too low to estimate. Values reported either in terms of means or medians depending on publication. *Unsp.* = unspecified or non-specific

Supplementary Table 1 (continued). FAHFAs reportedly present in human milk samples

FAHFA species/molecular species	Timepoint of sample collection	Mode of delivery	Concentration	Ref.
FAHFA 18:1/5O(FA 18:1)	*Unsp.*	*Unsp.*	*Unsp.*	(5)
FAHFA 18:1/6O(FA 18:1)	*Unsp.*	*Unsp.*	*Unsp.*	(5)
FAHFA 18:1/7O(FA 18:1)	*Unsp.*	*Unsp.*	*Unsp.*	(5)
FAHFA 18:1/8O(FA 18:1)	*Unsp.*	*Unsp.*	*Unsp.*	(5)
FAHFA 18:1/9O(FA 18:1)	*Unsp.*	*Unsp.*	*Unsp.*	(5)
FAHFA 18:1/18:2;O	Multiple timepoints	*Unsp.*	*Unsp.*	(2, 4, 5)
FAHFA 18:1/1O(FA 18:2)	*Unsp.*	*Unsp.*	*Unsp.*	(5)
FAHFA 18:1/2O(FA 18:2)	*Unsp.*	*Unsp.*	*Unsp.*	(5)
FAHFA 18:1/3O(FA 18:2)	*Unsp.*	*Unsp.*	*Unsp.*	(5)
FAHFA 18:1/4O(FA 18:2)	*Unsp.*	*Unsp.*	*Unsp.*	(5)
FAHFA 18:1/5O(FA 18:2)	*Unsp.*	*Unsp.*	*Unsp.*	(5)
FAHFA 18:1/6O(FA 18:2)	*Unsp.*	*Unsp.*	*Unsp.*	(5)
FAHFA 18:1/7O(FA 18:2)	*Unsp.*	*Unsp.*	*Unsp.*	(5)
FAHFA 18:1/8O(FA 18:2)	*Unsp.*	*Unsp.*	*Unsp.*	(5)
FAHFA 18:1/9O(FA 18:2)	*Unsp.*	*Unsp.*	*Unsp.*	(5)
FAHFA 18:1/20:0;O	Multiple timepoints	*Unsp.*	*Unsp.*	(5)
FAHFA 18:1/1O(FA 20:0)	*Unsp.*	*Unsp.*	*Unsp.*	(5)
FAHFA 18:1/2O(FA 20:0)	*Unsp.*	*Unsp.*	*Unsp.*	(5)

Values marked with ([1]) were estimated based on results presented in bar charts from (6) and line graphs from (7). Blank values marked with (*) were too low to estimate. Values reported either in terms of means or medians depending on publication. *Unsp.* = unspecified or non-specific

Supplementary Table 1 (continued). FAHFAs reportedly present in human milk samples

FAHFA species/molecular species	Timepoint of sample collection	Mode of delivery	Concentration	Ref.
FAHFA 18:1/20:3;O	≤ 72 hours, 1 month	*Unsp.*	*Unsp.*	(2, 3)
FAHFA 18:1/20:4;O	Multiple timepoints	*Unsp.*	*Unsp.*	(2)
FAHFA 18:1/22:4;O	Multiple timepoints	*Unsp.*	*Unsp.*	(2)
FAHFA 18:1/22:5;O	Multiple timepoints	*Unsp.*	*Unsp.*	(2)
FAHFA 18:1/24:4;O	Multiple timepoints	*Unsp.*	*Unsp.*	(2)
FAHFA 18:2/8:0;O	Multiple timepoints	*Unsp.*	*Unsp.*	(2)
FAHFA 18:2/10:0;O	Multiple timepoints	*Unsp.*	*Unsp.*	(2)
FAHFA 18:2/12:0;O	Multiple timepoints	*Unsp.*	*Unsp.*	(2, 5)
FAHFA 18:2/1O(FA 12:0)	*Unsp.*	*Unsp.*	*Unsp.*	(5)
FAHFA 18:2/2O(FA 12:0)	*Unsp.*	*Unsp.*	*Unsp.*	(5)
FAHFA 18:2/3O(FA 12:0)	*Unsp.*	*Unsp.*	*Unsp.*	(5)
FAHFA 18:2/4O(FA 12:0)	*Unsp.*	*Unsp.*	*Unsp.*	(5)
FAHFA 18:2/5O(FA 12:0)	*Unsp.*	*Unsp.*	*Unsp.*	(5)
FAHFA 18:2/6O(FA 12:0)	*Unsp.*	*Unsp.*	*Unsp.*	(5)
FAHFA 18:2/14:0;O	Multiple timepoints	*Unsp.*	*Unsp.*	(2, 4, 5)
FAHFA 18:2/1O(FA 14:0)	*Unsp.*	*Unsp.*	*Unsp.*	(5)
FAHFA 18:2/2O(FA 14:0)	*Unsp.*	*Unsp.*	*Unsp.*	(5)
FAHFA 18:2/3O(FA 14:0)	*Unsp.*	*Unsp.*	*Unsp.*	(5)

Values marked with ([1]) were estimated based on results presented in bar charts from (6) and line graphs from (7). Blank values marked with (*) were too low to estimate. Values reported either in terms of means or medians depending on publication. *Unsp.* = unspecified or non-specific

Supplementary Table 1 (continued). FAHFAs reportedly present in human milk samples

FAHFA species/molecular species	Timepoint of sample collection	Mode of delivery	Concentration	Ref.
FAHFA 18:2/4O(FA 14:0)	*Unsp.*	*Unsp.*	*Unsp.*	(5)
FAHFA 18:2/5O(FA 14:0)	*Unsp.*	*Unsp.*	*Unsp.*	(5)
FAHFA 18:2/6O(FA 14:0)	*Unsp.*	*Unsp.*	*Unsp.*	(5)
FAHFA 18:2/7O(FA 14:0)	*Unsp.*	*Unsp.*	*Unsp.*	(5)
FAHFA 18:2/8O(FA 14:0)	*Unsp.*	*Unsp.*	*Unsp.*	(5)
FAHFA 18:2/14:1;O	Multiple timepoints	*Unsp.*	*Unsp.*	(2, 5)
FAHFA 18:2/1O(FA 14:1)	*Unsp.*	*Unsp.*	*Unsp.*	(5)
FAHFA 18:2/2O(FA 14:1)	*Unsp.*	*Unsp.*	*Unsp.*	(5)
FAHFA 18:2/3O(FA 14:1)	*Unsp.*	*Unsp.*	*Unsp.*	(5)
FAHFA 18:2/14:2;O	Multiple timepoints	*Unsp.*	*Unsp.*	(5)
FAHFA 18:2/1O(FA 14:2)	*Unsp.*	*Unsp.*	*Unsp.*	(5)
FAHFA 18:2/2O(FA 14:2)	*Unsp.*	*Unsp.*	*Unsp.*	(5)
FAHFA 18:2/15:0;O	Multiple timepoints	*Unsp.*	*Unsp.*	(5)
FAHFA 18:2/1O(FA 15:0)	*Unsp.*	*Unsp.*	*Unsp.*	(5)
FAHFA 18:2/2O(FA 15:0)	*Unsp.*	*Unsp.*	*Unsp.*	(5)
FAHFA 18:2/3O(FA 15:0)	*Unsp.*	*Unsp.*	*Unsp.*	(5)
FAHFA 18:2/4O(FA 15:0)	*Unsp.*	*Unsp.*	*Unsp.*	(5)
FAHFA 18:2/5O(FA 15:0)	*Unsp.*	*Unsp.*	*Unsp.*	(5)

Values marked with ([1]) were estimated based on results presented in bar charts from (6) and line graphs from (7). Blank values marked with (*) were too low to estimate. Values reported either in terms of means or medians depending on publication. *Unsp.* = unspecified or non-specific

Supplementary Table 1 (continued). FAHFAs reportedly present in human milk samples

FAHFA species/molecular species	Timepoint of sample collection	Mode of delivery	Concentration	Ref.
FAHFA 18:2/6O(FA 15:0)	*Unsp.*	*Unsp.*	*Unsp.*	(5)
FAHFA 18:2/16:0;O	Multiple timepoints	*Unsp.*	*Unsp.*	(2, 4, 5)
FAHFA 18:2/1O(FA 16:0)	*Unsp.*	*Unsp.*	*Unsp.*	(5)
FAHFA 18:2/2O(FA 16:0)	*Unsp.*	*Unsp.*	*Unsp.*	(5)
FAHFA 18:2/3O(FA 16:0)	*Unsp.*	*Unsp.*	*Unsp.*	(5)
FAHFA 18:2/4O(FA 16:0)	*Unsp.*	*Unsp.*	*Unsp.*	(5)
FAHFA 18:2/5O(FA 16:0)	*Unsp.*	*Unsp.*	*Unsp.*	(5)
FAHFA 18:2/6O(FA 16:0)	*Unsp.*	*Unsp.*	*Unsp.*	(5)
FAHFA 18:2/7O(FA 16:0)	*Unsp.*	*Unsp.*	*Unsp.*	(5)
FAHFA 18:2/16:1;O	Multiple timepoints	*Unsp.*	*Unsp.*	(4, 5)
FAHFA 18:2/1O(FA 16:1)	*Unsp.*	*Unsp.*	*Unsp.*	(5)
FAHFA 18:2/2O(FA 16:1)	*Unsp.*	*Unsp.*	*Unsp.*	(5)
FAHFA 18:2/3O(FA 16:1)	*Unsp.*	*Unsp.*	*Unsp.*	(5)
FAHFA 18:2/4O(FA 16:1)	*Unsp.*	*Unsp.*	*Unsp.*	(5)
FAHFA 18:2/5O(FA 16:1)	*Unsp.*	*Unsp.*	*Unsp.*	(5)
FAHFA 18:2/6O(FA 16:1)	*Unsp.*	*Unsp.*	*Unsp.*	(5)
FAHFA 18:2/7O(FA 16:1)	*Unsp.*	*Unsp.*	*Unsp.*	(5)
FAHFA 18:2/8O(FA 16:1)	*Unsp.*	*Unsp.*	*Unsp.*	(5)

Values marked with ([1]) were estimated based on results presented in bar charts from (6) and line graphs from (7). Blank values marked with (*) were too low to estimate. Values reported either in terms of means or medians depending on publication. *Unsp.* = unspecified or non-specific

Supplementary Table 1 (continued). FAHFAs reportedly present in human milk samples

FAHFA species/molecular species	Timepoint of sample collection	Mode of delivery	Concentration	Ref.
FAHFA 18:2/9O(FA 16:1)	*Unsp.*	*Unsp.*	*Unsp.*	(5)
FAHFA 18:2/16:2;O	Multiple timepoints	*Unsp.*	*Unsp.*	(2, 5)
FAHFA 18:2/1O(FA 16:2)	*Unsp.*	*Unsp.*	*Unsp.*	(5)
FAHFA 18:2/2O(FA 16:2)	*Unsp.*	*Unsp.*	*Unsp.*	(5)
FAHFA 18:2/3O(FA 16:2)	*Unsp.*	*Unsp.*	*Unsp.*	(5)
FAHFA 18:2/4O(FA 16:2)	*Unsp.*	*Unsp.*	*Unsp.*	(5)
FAHFA 18:2/5O(FA 16:2)	*Unsp.*	*Unsp.*	*Unsp.*	(5)
FAHFA 18:2/17:0;O	Multiple timepoints	*Unsp.*	*Unsp.*	(5)
FAHFA 18:2/1O(FA 17:0)	*Unsp.*	*Unsp.*	*Unsp.*	(5)
FAHFA 18:2/18:0;O	Multiple timepoints	*Unsp.*	*Unsp.*	(4, 5)
FAHFA 18:2/1O(FA 18:0)	*Unsp.*	*Unsp.*	*Unsp.*	(5)
FAHFA 18:2/2O(FA 18:0)	*Unsp.*	*Unsp.*	*Unsp.*	(5)
FAHFA 18:2/3O(FA 18:0)	*Unsp.*	*Unsp.*	*Unsp.*	(5)
FAHFA 18:2/4O(FA 18:0)	*Unsp.*	*Unsp.*	*Unsp.*	(5)
FAHFA 18:2/5O(FA 18:0)	*Unsp.*	*Unsp.*	*Unsp.*	(5)
FAHFA 18:2/6O(FA 18:0)	*Unsp.*	*Unsp.*	*Unsp.*	(5)
FAHFA 18:2/7O(FA 18:0)	*Unsp.*	*Unsp.*	*Unsp.*	(5)
FAHFA 18:2/8O(FA 18:0)	*Unsp.*	*Unsp.*	*Unsp.*	(5)

Values marked with ([1]) were estimated based on results presented in bar charts from (6) and line graphs from (7). Blank values marked with (*) were too low to estimate. Values reported either in terms of means or medians depending on publication. *Unsp.* = unspecified or non-specific

Supplementary Table 1 (continued). FAHFAs reportedly present in human milk samples

FAHFA species/molecular species	Timepoint of sample collection	Mode of delivery	Concentration	Ref.
FAHFA 18:2/18:1;O	Multiple timepoints	*Unsp.*	*Unsp.*	(2-5)
FAHFA 18:2/1O(FA 18:1)	*Unsp.*	*Unsp.*	*Unsp.*	(5)
FAHFA 18:2/2O(FA 18:1)	*Unsp.*	*Unsp.*	*Unsp.*	(5)
FAHFA 18:2/3O(FA 18:1)	*Unsp.*	*Unsp.*	*Unsp.*	(5)
FAHFA 18:2/4O(FA 18:1)	*Unsp.*	*Unsp.*	*Unsp.*	(5)
FAHFA 18:2/5O(FA 18:1)	*Unsp.*	*Unsp.*	*Unsp.*	(5)
FAHFA 18:2/6O(FA 18:1)	*Unsp.*	*Unsp.*	*Unsp.*	(5)
FAHFA 18:2/18:2;O	Multiple timepoints	*Unsp.*	0.03 µmol/L at 1 month	(1, 2, 4, 5)
FAHFA 18:2/1O(FA 18:2)	*Unsp.*	*Unsp.*	*Unsp.*	(5)
FAHFA 18:2/2O(FA 18:2)	*Unsp.*	*Unsp.*	*Unsp.*	(5)
FAHFA 18:2/3O(FA 18:2)	*Unsp.*	*Unsp.*	*Unsp.*	(5)
FAHFA 18:2/4O(FA 18:2)	*Unsp.*	*Unsp.*	*Unsp.*	(5)
FAHFA 18:2/5O(FA 18:2)	*Unsp.*	*Unsp.*	*Unsp.*	(5)
FAHFA 18:2/6O(FA 18:2)	*Unsp.*	*Unsp.*	*Unsp.*	(5)
FAHFA 18:2/7O(FA 18:2)	*Unsp.*	*Unsp.*	*Unsp.*	(5)

Values marked with ([1]) were estimated based on results presented in bar charts from (6) and line graphs from (7). Blank values marked with (*) were too low to estimate. Values reported either in terms of means or medians depending on publication. *Unsp.* = unspecified or non-specific

Supplementary Table 1 (continued). FAHFAs reportedly present in human milk samples

FAHFA species/molecular species	Timepoint of sample collection	Mode of delivery	Concentration	Ref.
FAHFA 18:2/13O(FA 18:2)	72 hours	Vaginal (38-41 weeks)	1.6 nmol/L[1]	(6)
	72 hours	Caesarean (38-41 weeks)	*	(6)
	72 hours	Before 32 weeks	*	(6)
	≤ 72 hours	*Unsp.*	0.9 nmol/L[1]<br>2.5 nmol/L[1] (BMI > 25)	(7)
	28 days	Vaginal (38-41 weeks)	18.0 nmol/L[1]	(6)
	28 days	Caesarean (38-41 weeks)	21.2 nmol/L[1]	(6)
	28 days	Before 32 weeks	22.1 nmol/L[1]	(6)
	42 days	*Unsp.*	20.6 nmol/L[1]<br>17.3 nmol/L[1] (BMI > 25)	(7)
	3 months	*Unsp.*	28.1 nmol/L[1]<br>20.6 nmol/L[1] (BMI > 25)	(7)
	36 weeks	Before 32 weeks	14.2 nmol/L[1]	(6)
FAHFA 18:2/20:0;O	Multiple timepoints	*Unsp.*	*Unsp.*	(5)
FAHFA 18:2/1O(FA 20:0)	*Unsp.*	*Unsp.*	*Unsp.*	(5)
FAHFA 18:2/2O(FA 20:0)	*Unsp.*	*Unsp.*	*Unsp.*	(5)
FAHFA 18:2/20:4;O	≤ 72 hours, 1 month	*Unsp.*	*Unsp.*	(2, 3)
FAHFA 18:2/22:4;O	Multiple timepoints	*Unsp.*	*Unsp.*	(2)
FAHFA 18:2/22:5;O	Multiple timepoints	*Unsp.*	*Unsp.*	(2)
FAHFA 18:3/12:0;O	Multiple timepoints	*Unsp.*	*Unsp.*	(5)
FAHFA 18:3/1O(FA 12:0)	*Unsp.*	*Unsp.*	*Unsp.*	(5)

Values marked with ([1]) were estimated based on results presented in bar charts from (6) and line graphs from (7). Blank values marked with (*) were too low to estimate. Values reported either in terms of means or medians depending on publication. *Unsp.* = unspecified or non-specific

Supplementary Table 1 (continued). FAHFAs reportedly present in human milk samples

FAHFA species/molecular species	Timepoint of sample collection	Mode of delivery	Concentration	Ref.
FAHFA 18:3/2O(FA 12:0)	*Unsp.*	*Unsp.*	*Unsp.*	(5)
FAHFA 18:3/3O(FA 12:0)	*Unsp.*	*Unsp.*	*Unsp.*	(5)
FAHFA 18:3/4O(FA 12:0)	*Unsp.*	*Unsp.*	*Unsp.*	(5)
FAHFA 18:3/14:0;O	Multiple timepoints	*Unsp.*	*Unsp.*	(5)
FAHFA 18:3/1O(FA 14:0)	*Unsp.*	*Unsp.*	*Unsp.*	(5)
FAHFA 18:3/2O(FA 14:0)	*Unsp.*	*Unsp.*	*Unsp.*	(5)
FAHFA 18:3/3O(FA 14:0)	*Unsp.*	*Unsp.*	*Unsp.*	(5)
FAHFA 18:3/4O(FA 14:0)	*Unsp.*	*Unsp.*	*Unsp.*	(5)
FAHFA 18:3/16:0;O	Multiple timepoints	*Unsp.*	*Unsp.*	(2, 4, 5)
FAHFA 18:3/1O(FA 16:0)	*Unsp.*	*Unsp.*	*Unsp.*	(5)
FAHFA 18:3/2O(FA 16:0)	*Unsp.*	*Unsp.*	*Unsp.*	(5)
FAHFA 18:3/3O(FA 16:0)	*Unsp.*	*Unsp.*	*Unsp.*	(5)
FAHFA 18:3/4O(FA 16:0)	*Unsp.*	*Unsp.*	*Unsp.*	(5)
FAHFA 18:3/5O(FA 16:0)	*Unsp.*	*Unsp.*	*Unsp.*	(5)
FAHFA 18:3/6O(FA 16:0)	*Unsp.*	*Unsp.*	*Unsp.*	(5)
FAHFA 18:3/7O(FA 16:0)	*Unsp.*	*Unsp.*	*Unsp.*	(5)
FAHFA 18:3/16:1;O	Multiple timepoints	*Unsp.*	*Unsp.*	(5)
FAHFA 18:3/1O(FA 16:1)	*Unsp.*	*Unsp.*	*Unsp.*	(5)

Values marked with ([1]) were estimated based on results presented in bar charts from (6) and line graphs from (7). Blank values marked with (*) were too low to estimate. Values reported either in terms of means or medians depending on publication. *Unsp.* = unspecified or non-specific

Supplementary Table 1 (continued). FAHFAs reportedly present in human milk samples

FAHFA species/molecular species	Timepoint of sample collection	Mode of delivery	Concentration	Ref.
FAHFA 18:3/2O(FA 16:1)	*Unsp.*	*Unsp.*	*Unsp.*	(5)
FAHFA 18:3/3O(FA 16:1)	*Unsp.*	*Unsp.*	*Unsp.*	(5)
FAHFA 18:3/4O(FA 16:1)	*Unsp.*	*Unsp.*	*Unsp.*	(5)
FAHFA 18:3/5O(FA 16:1)	*Unsp.*	*Unsp.*	*Unsp.*	(5)
FAHFA 18:3/6O(FA 16:1)	*Unsp.*	*Unsp.*	*Unsp.*	(5)
FAHFA 18:3/16:2;O	Multiple timepoints	*Unsp.*	*Unsp.*	(5)
FAHFA 18:3/1O(FA 16:2)	*Unsp.*	*Unsp.*	*Unsp.*	(5)
FAHFA 18:3/2O(FA 16:2)	*Unsp.*	*Unsp.*	*Unsp.*	(5)
FAHFA 18:3/3O(FA 16:2)	*Unsp.*	*Unsp.*	*Unsp.*	(5)
FAHFA 18:3/18:0;O	Multiple timepoints	*Unsp.*	*Unsp.*	(5)
FAHFA 18:3/1O(FA 18:0)	*Unsp.*	*Unsp.*	*Unsp.*	(5)
FAHFA 18:3/2O(FA 18:0)	*Unsp.*	*Unsp.*	*Unsp.*	(5)
FAHFA 18:3/18:1;O	Pooled 6 weeks, 3 months, 6 months	*Unsp.*	*Unsp.*	(4)
FAHFA 18:3/18:2;O	Multiple timepoints	*Unsp.*	*Unsp.*	(2)
FAHFA 18:3/22:5;O	Multiple timepoints	*Unsp.*	*Unsp.*	(2)
FAHFA 20:1/20:0;O	Multiple timepoints	*Unsp.*	*Unsp.*	(2)
FAHFA 20:1/20:2;O	Multiple timepoints	*Unsp.*	*Unsp.*	(2)
FAHFA 20:1/20:3;O	Multiple timepoints	*Unsp.*	*Unsp.*	(2)

Values marked with ([1]) were estimated based on results presented in bar charts from (6) and line graphs from (7). Blank values marked with (*) were too low to estimate. Values reported either in terms of means or medians depending on publication. *Unsp.* = unspecified or non-specific

Supplementary Table 1 (continued). FAHFAs reportedly present in human milk samples

FAHFA species/molecular species	Timepoint of sample collection	Mode of delivery	Concentration	Ref.
FAHFA 20:2/18:0;O	Multiple timepoints	*Unsp.*	*Unsp.*	(2)
FAHFA 20:2/20:1;O	Multiple timepoints	*Unsp.*	*Unsp.*	(2)
FAHFA 20:2/21:2;O	Multiple timepoints	*Unsp.*	*Unsp.*	(2)
FAHFA 20:2/22:4;O	Multiple timepoints	*Unsp.*	*Unsp.*	(2)
FAHFA 20:3/16:0;O	Multiple timepoints	*Unsp.*	*Unsp.*	(5)
FAHFA 20:3/1O(FA 16:0)	*Unsp.*	*Unsp.*	*Unsp.*	(5)
FAHFA 20:3/2O(FA 16:0)	*Unsp.*	*Unsp.*	*Unsp.*	(5)
FAHFA 20:3/3O(FA 16:0)	*Unsp.*	*Unsp.*	*Unsp.*	(5)
FAHFA 20:3/4O(FA 16:0)	*Unsp.*	*Unsp.*	*Unsp.*	(5)
FAHFA 20:3/5O(FA 16:0)	*Unsp.*	*Unsp.*	*Unsp.*	(5)
FAHFA 20:3/6O(FA 16:0)	*Unsp.*	*Unsp.*	*Unsp.*	(5)
FAHFA 20:3/16:1;O	Multiple timepoints	*Unsp.*	*Unsp.*	(5)
FAHFA 20:3/1O(FA 16:1)	*Unsp.*	*Unsp.*	*Unsp.*	(5)
FAHFA 20:3/2O(FA 16:1)	*Unsp.*	*Unsp.*	*Unsp.*	(5)
FAHFA 20:3/3O(FA 16:1)	*Unsp.*	*Unsp.*	*Unsp.*	(5)
FAHFA 20:3/18:2;O	Multiple timepoints	*Unsp.*	*Unsp.*	(5)
FAHFA 20:3/1O(FA 18:2)	*Unsp.*	*Unsp.*	*Unsp.*	(5)
FAHFA 20:3/20:2;O	Multiple timepoints	*Unsp.*	*Unsp.*	(2)

Values marked with ([1]) were estimated based on results presented in bar charts from (6) and line graphs from (7). Blank values marked with (*) were too low to estimate. Values reported either in terms of means or medians depending on publication. *Unsp.* = unspecified or non-specific

Supplementary Table 1 (continued). FAHFAs reportedly present in human milk samples

FAHFA species/molecular species	Timepoint of sample collection	Mode of delivery	Concentration	Ref.
FAHFA 20:3/20:4;O	Multiple timepoints	*Unsp.*	*Unsp.*	(2)
FAHFA 20:3/22:5;O	Multiple timepoints	*Unsp.*	*Unsp.*	(2)
FAHFA 20:4/14:0;O	Multiple timepoints	*Unsp.*	*Unsp.*	(5)
FAHFA 20:4/1O(FA 14:0)	*Unsp.*	*Unsp.*	*Unsp.*	(5)
FAHFA 20:4/2O(FA 14:0)	*Unsp.*	*Unsp.*	*Unsp.*	(5)
FAHFA 20:4/3O(FA 14:0)	*Unsp.*	*Unsp.*	*Unsp.*	(5)
FAHFA 20:4/4O(FA 14:0)	*Unsp.*	*Unsp.*	*Unsp.*	(5)
FAHFA 20:4/16:0;O	Multiple timepoints	*Unsp.*	*Unsp.*	(2, 5)
FAHFA 20:4/1O(FA 16:0)	*Unsp.*	*Unsp.*	*Unsp.*	(5)
FAHFA 20:4/2O(FA 16:0)	*Unsp.*	*Unsp.*	*Unsp.*	(5)
FAHFA 20:4/3O(FA 16:0)	*Unsp.*	*Unsp.*	*Unsp.*	(5)
FAHFA 20:4/4O(FA 16:0)	*Unsp.*	*Unsp.*	*Unsp.*	(5)
FAHFA 20:4/5O(FA 16:0)	*Unsp.*	*Unsp.*	*Unsp.*	(5)
FAHFA 20:4/6O(FA 16:0)	*Unsp.*	*Unsp.*	*Unsp.*	(5)
FAHFA 20:4/7O(FA 16:0)	*Unsp.*	*Unsp.*	*Unsp.*	(5)
FAHFA 20:4/16:1;O	Multiple timepoints	*Unsp.*	*Unsp.*	(5)
FAHFA 20:4/1O(FA 16:1)	*Unsp.*	*Unsp.*	*Unsp.*	(5)
FAHFA 20:4/2O(FA 16:1)	*Unsp.*	*Unsp.*	*Unsp.*	(5)

Values marked with ([1]) were estimated based on results presented in bar charts from (6) and line graphs from (7). Blank values marked with (*) were too low to estimate. Values reported either in terms of means or medians depending on publication. *Unsp.* = unspecified or non-specific

Supplementary Table 1 (continued). FAHFAs reportedly present in human milk samples

FAHFA species/molecular species	Timepoint of sample collection	Mode of delivery	Concentration	Ref.
FAHFA 20:4/3O(FA 16:1)	*Unsp.*	*Unsp.*	*Unsp.*	(5)
FAHFA 20:4/4O(FA 16:1)	*Unsp.*	*Unsp.*	*Unsp.*	(5)
FAHFA 20:4/5O(FA 16:1)	*Unsp.*	*Unsp.*	*Unsp.*	(5)
FAHFA 20:4/16:2;O	Multiple timepoints	*Unsp.*	*Unsp.*	(5)
FAHFA 20:4/1O(FA 16:2)	*Unsp.*	*Unsp.*	*Unsp.*	(5)
FAHFA 20:4/2O(FA 16:2)	*Unsp.*	*Unsp.*	*Unsp.*	(5)
FAHFA 20:4/3O(FA 16:2)	*Unsp.*	*Unsp.*	*Unsp.*	(5)
FAHFA 20:4/18:0;O	Multiple timepoints	*Unsp.*	*Unsp.*	(5)
FAHFA 20:4/1O(FA 18:0)	*Unsp.*	*Unsp.*	*Unsp.*	(5)
FAHFA 20:4/2O(FA 18:0)	*Unsp.*	*Unsp.*	*Unsp.*	(5)
FAHFA 20:4/18:1;O	Multiple timepoints	*Unsp.*	*Unsp.*	(2, 5)
FAHFA 20:4/1O(FA 18:1)	*Unsp.*	*Unsp.*	*Unsp.*	(5)
FAHFA 20:4/18:2;O	Multiple timepoints	*Unsp.*	*Unsp.*	(2, 5)
FAHFA 20:4/1O(FA 18:2)	*Unsp.*	*Unsp.*	*Unsp.*	(5)
FAHFA 20:4/2O(FA 18:2)	*Unsp.*	*Unsp.*	*Unsp.*	(5)
FAHFA 20:4/3O(FA 18:2)	*Unsp.*	*Unsp.*	*Unsp.*	(5)
FAHFA 20:4/19:1;O	Multiple timepoints	*Unsp.*	*Unsp.*	(2)
FAHFA 20:4/20:3;O	≤ 72 hours, 1 month	*Unsp.*	*Unsp.*	(2, 3)

Values marked with ([1]) were estimated based on results presented in bar charts from (6) and line graphs from (7). Blank values marked with (*) were too low to estimate. Values reported either in terms of means or medians depending on publication. *Unsp.* = unspecified or non-specific

Supplementary Table 1 (continued). FAHFAs reportedly present in human milk samples

FAHFA species/molecular species	Timepoint of sample collection	Mode of delivery	Concentration	Ref.
FAHFA 20:5/22:0;O	≤ 72 hours	*Unsp.*	*Unsp.*	(3)
FAHFA 20:5/24:2;O	≤ 72 hours	*Unsp.*	*Unsp.*	(3)
FAHFA 21:0/18:3;O	≤ 72 hours	*Unsp.*	*Unsp.*	(3)
FAHFA 21:0/20:4;O	Multiple timepoints	*Unsp.*	*Unsp.*	(2)
FAHFA 22:1/24:4;O	Multiple timepoints	*Unsp.*	*Unsp.*	(2)
FAHFA 22:2/24:4;O	Multiple timepoints	*Unsp.*	*Unsp.*	(2)
FAHFA 22:3/22:4;O	Multiple timepoints	*Unsp.*	*Unsp.*	(2)
FAHFA 22:4/11:0;O	Multiple timepoints	*Unsp.*	*Unsp.*	(2)
FAHFA 22:4/20:3;O	Multiple timepoints	*Unsp.*	*Unsp.*	(2)
FAHFA 22:4/21:2;O	Multiple timepoints	*Unsp.*	*Unsp.*	(2)
FAHFA 22:4/22:3;O	Multiple timepoints	*Unsp.*	*Unsp.*	(2)
FAHFA 22:5/20:4;O	Multiple timepoints	*Unsp.*	*Unsp.*	(2)
FAHFA 22:5/26:1;O	Multiple timepoints	*Unsp.*	*Unsp.*	(2)
FAHFA 22:6/22:2;O	Multiple timepoints	*Unsp.*	*Unsp.*	(2)
FAHFA 22:6/22:5;O	≤ 72 hours, 1 month	*Unsp.*	*Unsp.*	(2, 3)
FAHFA 22:6/24:1;O	Multiple timepoints	*Unsp.*	*Unsp.*	(2)
FAHFA 24:1/20:4;O	Multiple timepoints	*Unsp.*	*Unsp.*	(2)
FAHFA 24:2/22:5;O	Multiple timepoints	*Unsp.*	*Unsp.*	(2)

Values marked with ([1]) were estimated based on results presented in bar charts from (6) and line graphs from (7). Blank values marked with (*) were too low to estimate. Values reported either in terms of means or medians depending on publication. *Unsp.* = unspecified or non-specific

Supplementary Table 1 (continued). FAHFAs reportedly present in human milk samples

FAHFA species/molecular species	Timepoint of sample collection	Mode of delivery	Concentration	Ref.
FAHFA 24:2/26:4;O	Multiple timepoints	*Unsp.*	*Unsp.*	(2)
FAHFA 24:4/20:0;O	Multiple timepoints	*Unsp.*	*Unsp.*	(2)
FAHFA 24:4/22:4;O	≤ 72 hours	*Unsp.*	*Unsp.*	(3)
FAHFA 26:2/20:4;O	≤ 72 hours, 1 month	*Unsp.*	*Unsp.*	(2, 3)
FAHFA 26:4/16:0;O	Multiple timepoints	*Unsp.*	*Unsp.*	(2)

Values marked with ([1]) were estimated based on results presented in bar charts from (6) and line graphs from (7). Blank values marked with (*) were too low to estimate. Values reported either in terms of means or medians depending on publication. *Unsp.* = unspecified or non-specific

Supplementary Table 2. TG(O)s reportedly present in human milk samples

TG(O) species/molecular species	Timepoint of sample collection	Concentration/proportion	Ref.
TG O-40:1	Pooled 3 months, 6 months	*Unsp.*	(4)
TG O-16:0_6:0_18:1	Pooled 3 months, 6 months	*Unsp.*	(4)
TG O-16:1_12:0_12:0	Pooled 3 months, 6 months	*Unsp.*	(4)
TG O-44:1	*Unsp.*	1.6%	(4, 8)
TG O-16:0_10:0_18:1	Pooled 3 months, 6 months	*Unsp.*	(4)
TG O-16:1_12:0_16:0	Pooled 6 weeks, 3 months, 6 months	*Unsp.*	(4)
TG O-18:1_10:0_16:0	Pooled 6 weeks, 3 months, 6 months	*Unsp.*	(4)
TG O-18:1/16:0/14:0	*Unsp.*	*Unsp.*	(8)
TG O-18:1/14:0/12:0	*Unsp.*	*Unsp.*	(8)
TG O-46:1	*Unsp.*	2.1%	(8)
TG O-16:0/18:1/12:0	*Unsp.*	*Unsp.*	(8)
TG O-18:0/18:1/10:0	*Unsp.*	*Unsp.*	(8)
TG O-18:1/12:0/16:0	*Unsp.*	*Unsp.*	(8)
TG O-48:0	1 month	4412.2 nmol/L	(9)
	6 months	4245.1 nmol/L	(9)
	12 months	5577.3 nmol/L	(9)
	Pooled 6 weeks, 3 months, 6 months	*Unsp.*	(4)
	Unsp.	1.2%	(8)

Unsp. = unspecified or non-specific

Supplementary Table 2 (continued). TG(O)s reportedly present in human milk samples

TG(O) species/molecular species	Timepoint of sample collection	Concentration/proportion	Ref.
TG O-16:0_16:0_16:0	Pooled 6 weeks, 3 months, 6 months	*Unsp.*	(4)
TG O-18:0_14:0_16:0	Pooled 6 weeks, 3 months, 6 months	*Unsp.*	(4)
TG O-20:0_12:0_16:0	Pooled 6 weeks, 3 months, 6 months	*Unsp.*	(4)
TG O-16:0/20:0/12:0	*Unsp.*	1.2%	(8)
TG O-48:0 [NL-16:0]	1 month	15949.2 nmol/L	(9)
	6 months	15358.1 nmol/L	(9)
	12 months	19079.7 nmol/L	(9)
TG O-48:1	1 month	3598.6 nmol/L	(9)
	6 months	3426.2 nmol/L	(9)
	12 months	4949.8 nmol/L	(9)
	Unsp.	2.2%	(8)
TG O-16:0/16:0/16:1	*Unsp.*	*Unsp.*	(8)
TG O-18:0/18:1/12:0	*Unsp.*	*Unsp.*	(8)
TG O-48:1 [NL-16:0]	1 month	6309.2 nmol/L	(9)
	6 months	5884.2 nmol/L	(9)
	12 months	7928.1 nmol/L	(9)
TG O-48:1 [NL-16:1]	1 month	817.3 nmol/L	(9)
	6 months	666.7 nmol/L	(9)
	12 months	832.5 nmol/L	(9)

Unsp. = unspecified or non-specific

Supplementary Table 2 (continued). TG(O)s reportedly present in human milk samples

TG(O) species/molecular species	Timepoint of sample collection	Concentration/proportion	Ref.
TG O-48:2	1 month	981.3 nmol/L	(9)
	6 months	1014.8 nmol/L	(9)
	12 months	1159.2 nmol/L	(9)
	Unsp.	1.9%	(8)
TG O-18:1/12:0/18:1	*Unsp.*	1.9%	(8)
TG O-48:2 [NL-16:0]	1 month	279.8 nmol/L	(9)
	6 months	232.7 nmol/L	(9)
	12 months	264.7 nmol/L	(9)
TG O-48:2 [NL-16:1]	1 month	138.5 nmol/L	(9)
	6 months	108.4 nmol/L	(9)
	12 months	140.8 nmol/L	(9)
TG O-48:3	1 month	1035.6 nmol/L	(9)
	6 months	1102.9 nmol/L	(9)
	12 months	1085.7 nmol/L	(9)
TG O-48:3 [NL-16:1]	1 month	31.4 nmol/L	(9)
	6 months	27.8 nmol/L	(9)
	12 months	18.8 nmol/L	(9)

Unsp. = unspecified or non-specific

Supplementary Table 2 (continued). TG(O)s reportedly present in human milk samples

TG(O) species/molecular species	Timepoint of sample collection	Concentration/proportion	Ref.
TG O-50:0	1 month	5766.3 nmol/L	(9)
	6 months	5230.5 nmol/L	(9)
	12 months	4915.0 nmol/L	(9)
	Pooled 6 weeks, 3 months, 6 months	*Unsp.*	(4)
	Unsp.	1.7%	(8)
TG O-16:0_16:0_18:0	Pooled 6 weeks, 3 months, 6 months	*Unsp.*	(4)
TG O-18:0_16:0_16:0	Pooled 6 weeks, 3 months, 6 months	*Unsp.*	(4)
TG O-20:0_14:0_16:0	Pooled 6 weeks, 3 months, 6 months	*Unsp.*	(4)
TG O-16:0/16:0/18:0	*Unsp.*	*Unsp.*	(8)
TG O-18:0/16:0/16:0	*Unsp.*	*Unsp.*	(8)
TG O-50:0 [NL-16:0]	1 month	19235.3 nmol/L	(9)
	6 months	16223.4 nmol/L	(9)
	12 months	14878.0 nmol/L	(9)
TG O-50:0 [NL-18:0]	1 month	6417.2 nmol/L	(9)
	6 months	5906.2 nmol/L	(9)
	12 months	5330.9 nmol/L	(9)

Unsp. = unspecified or non-specific

Supplementary Table 2 (continued). TG(O)s reportedly present in human milk samples

TG(O) species/molecular species	Timepoint of sample collection	Concentration/proportion	Ref.
TG O-50:1	1 month	25063.7 nmol/L	(9)
	6 months	24661.7 nmol/L	(9)
	12 months	28311.3 nmol/L	(9)
	Multiple timepoints	*Unsp.*	(4, 10)
	Unsp.	8.9%	(8)
TG O-16:0_16:0_18:1	Pooled 6 weeks, 3 months, 6 months	*Unsp.*	(4)
TG O-18:1_16:0_16:0	Pooled 6 weeks, 3 months, 6 months	*Unsp.*	(4)
TG O-16:0/16:0/18:1	*Unsp.*	*Unsp.*	(8)
TG O-18:0/16:0/16:1	*Unsp.*	*Unsp.*	(8)
TG O-18:1/16:0/16:0	*Unsp.*	*Unsp.*	(8)
TG O-50:1 [NL-16:0]	1 month	86059.9 nmol/L	(9)
	6 months	85013.4 nmol/L	(9)
	12 months	92836.1 nmol/L	(9)
TG O-50:1 [NL-18:1]	1 month	43434.6 nmol/L	(9)
	6 months	43050.7 nmol/L	(9)
	12 months	50312.8 nmol/L	(9)
TG O-50:1 [NL-O-16:0]	1 month	7610.0 nmol/L	(9)
	6 months	7345.8 nmol/L	(9)
	12 months	8665.5 nmol/L	(9)

Unsp. = unspecified or non-specific

Supplementary Table 2 (continued). TG(O)s reportedly present in human milk samples

TG(O) species/molecular species	Timepoint of sample collection	Concentration/proportion	Ref.
TG O-50:1 [NL-O-18:0]	1 month	3436.1 nmol/L	(9)
	6 months	3904.2 nmol/L	(9)
	12 months	4188.4 nmol/L	(9)
TG O-50:1 [NL-O-18:1]	1 month	5801.2 nmol/L	(9)
	6 months	5217.2 nmol/L	(9)
	12 months	6184.3 nmol/L	(9)
TG O-50:2	1 month	4500.7 nmol/L	(9)
	6 months	3920.1 nmol/L	(9)
	12 months	5080.0 nmol/L	(9)
	Unsp.	4.5%	(8)
TG O-16:0/16:0/18:2	*Unsp.*	*Unsp.*	(8)
TG O-16:0/18:1/16:1	*Unsp.*	*Unsp.*	(8)
TG O-18:1/16:0/16:1	*Unsp.*	*Unsp.*	(8)
TG O-18:2/16:0/16:0	*Unsp.*	*Unsp.*	(8)
TG O-50:2 [NL-16:0]	1 month	7423.3 nmol/L	(9)
	6 months	5457.9 nmol/L	(9)
	12 months	6655.0 nmol/L	(9)

Unsp. = unspecified or non-specific

Supplementary Table 2 (continued). TG(O)s reportedly present in human milk samples

TG(O) species/molecular species	Timepoint of sample collection	Concentration/proportion	Ref.
TG O-50:2 [NL-16:1]	1 month	3456.8 nmol/L	(9)
	6 months	2402.5 nmol/L	(9)
	12 months	2647.9 nmol/L	(9)
TG O-50:2 [NL-18:1]	1 month	6039.5 nmol/L	(9)
	6 months	5505.5 nmol/L	(9)
	12 months	6648.3 nmol/L	(9)
TG O-50:2 [NL-18:2]	1 month	2513.5 nmol/L	(9)
	6 months	2313.0 nmol/L	(9)
	12 months	3190.0 nmol/L	(9)
TG O-50:2 [NL-O-16:0]	1 month	663.1 nmol/L	(9)
	6 months	562.3 nmol/L	(9)
	12 months	720.8 nmol/L	(9)
TG O-50:2 [NL-O-18:0]	1 month	158.6 nmol/L	(9)
	6 months	171.5 nmol/L	(9)
	12 months	219.1 nmol/L	(9)
TG O-50:2 [NL-O-18:1]	1 month	1992.1 nmol/L	(9)
	6 months	1853.0 nmol/L	(9)
	12 months	2227.6 nmol/L	(9)

Unsp. = unspecified or non-specific

Supplementary Table 2 (continued). TG(O)s reportedly present in human milk samples

TG(O) species/molecular species	Timepoint of sample collection	Concentration/proportion	Ref.
TG O-50:3	1 month	704.4 nmol/L	(9)
	6 months	657.0 nmol/L	(9)
	12 months	735.6 nmol/L	(9)
TG O-50:3 [NL-18:2]	1 month	311.1 nmol/L	(9)
	6 months	320.9 nmol/L	(9)
	12 months	349.2 nmol/L	(9)
TG O-50:4	1 month	4045.4 nmol/L	(9)
	6 months	3322.0 nmol/L	(9)
	12 months	3261.0 nmol/L	(9)
TG O-50:4 [NL-16:0]	1 month	8.0 nmol/L	(9)
	6 months	7.5 nmol/L	(9)
	12 months	6.3 nmol/L	(9)
TG O-50:4 [NL-16:1]	1 month	23.9 nmol/L	(9)
	6 months	19.8 nmol/L	(9)
	12 months	15.7 nmol/L	(9)
TG O-50:4 [NL-18:3]	1 month	90.4 nmol/L	(9)
	6 months	97.8 nmol/L	(9)
	12 months	101.3 nmol/L	(9)

Unsp. = unspecified or non-specific

Supplementary Table 2 (continued). TG(O)s reportedly present in human milk samples

TG(O) species/molecular species	Timepoint of sample collection	Concentration/proportion	Ref.
TG O-52:0	1 month	1450.8 nmol/L	(9)
	6 months	1264.3 nmol/L	(9)
	12 months	1087.9 nmol/L	(9)
	Pooled 6 weeks, 3 months, 6 months	*Unsp.*	(4)
TG O-16:0_16:0_20:0	Pooled 6 weeks, 3 months, 6 months	*Unsp.*	(4)
TG O-18:0_16:0_18:0	Pooled 6 weeks, 3 months, 6 months	*Unsp.*	(4)
TG O-20:0_16:0_16:0	Pooled 6 weeks, 3 months, 6 months	*Unsp.*	(4)
TG O-52:0 [NL-16:0]	1 month	4764.2 nmol/L	(9)
	6 months	4017.1 nmol/L	(9)
	12 months	3224.6 nmol/L	(9)
TG O-52:0 [NL-18:0]	1 month	2932.4 nmol/L	(9)
	6 months	2473.7 nmol/L	(9)
	12 months	1847.8 nmol/L	(9)
TG O-52:0 [NL-O-16:0]	1 month	199.3 nmol/L	(9)
	6 months	152.8 nmol/L	(9)
	12 months	124.0 nmol/L	(9)
TG O-52:0 [NL-O-18:0]	1 month	850.5 nmol/L	(9)
	6 months	725.6 nmol/L	(9)
	12 months	545.3 nmol/L	(9)

Unsp. = unspecified or non-specific

Supplementary Table 2 (continued). TG(O)s reportedly present in human milk samples

TG(O) species/molecular species	Timepoint of sample collection	Concentration/proportion	Ref.
TG O-52:1	1 month	25942.9 nmol/L	(9)
	6 months	24715.0 nmol/L	(9)
	12 months	20875.9 nmol/L	(9)
	Multiple timepoints	*Unsp.*	(4, 10)
	Unsp.	10.8%	(8)
TG O-18:0_16:0_18:1	Pooled 6 weeks, 3 months, 6 months	*Unsp.*	(4)
TG O-18:1_16:0_18:0	Pooled 6 weeks, 3 months, 6 months	*Unsp.*	(4)
TG O-20:1_16:0_16:0	Pooled 6 weeks, 3 months, 6 months	*Unsp.*	(4)
TG O-16:0/18:1/18:0	*Unsp.*	*Unsp.*	(8)
TG O-18:0/16:0/18:1	*Unsp.*	*Unsp.*	(8)
TG O-18:1/16:0/18:0	*Unsp.*	*Unsp.*	(8)
TG O-52:1 [NL-16:0]	1 month	89734.2 nmol/L	(9)
	6 months	89264.9 nmol/L	(9)
	12 months	71874.1 nmol/L	(9)
TG O-52:1 [NL-18:0]	1 month	21754.3 nmol/L	(9)
	6 months	19457.0 nmol/L	(9)
	12 months	16238.8 nmol/L	(9)

Unsp. = unspecified or non-specific

Supplementary Table 2 (continued). TG(O)s reportedly present in human milk samples

TG(O) species/molecular species	Timepoint of sample collection	Concentration/proportion	Ref.
TG O-52:1 [NL-18:1]	1 month	54675.1 nmol/L	(9)
	6 months	51429.4 nmol/L	(9)
	12 months	43882.9 nmol/L	(9)
TG O-52:1 [NL-O-16:0]	1 month	2407.8 nmol/L	(9)
	6 months	1997.5 nmol/L	(9)
	12 months	1856.2 nmol/L	(9)
TG O-52:1 [NL-O-18:0]	1 month	14495.7 nmol/L	(9)
	6 months	14214.7 nmol/L	(9)
	12 months	11943.6 nmol/L	(9)
TG O-52:1 [NL-O-18:1]	1 month	3694.2 nmol/L	(9)
	6 months	3746.5 nmol/L	(9)
	12 months	3063.8 nmol/L	(9)
TG O-52:2	1 month	47451.3 nmol/L	(9)
	6 months	42384.6 nmol/L	(9)
	12 months	40973.1 nmol/L	(9)
	Multiple timepoints	*Unsp.*	(4, 10)
	Unsp.	25.3%	(8)
TG O-16:0_18:1_18:1	Pooled 6 weeks, 3 months, 6 months	*Unsp.*	(4)
TG O-18:1_16:0_18:1	Pooled 6 weeks, 3 months, 6 months	*Unsp.*	(4)

Unsp. = unspecified or non-specific

Supplementary Table 2 (continued). TG(O)s reportedly present in human milk samples

TG(O) species/molecular species	Timepoint of sample collection	Concentration/proportion	Ref.
TG O-16:0/18:2/18:0	*Unsp.*	*Unsp.*	(8)
TG O-18:0/16:0/18:2	*Unsp.*	*Unsp.*	(8)
TG O-18:1/16:0/18:1	*Unsp.*	*Unsp.*	(8)
TG O-52:2 [NL-16:0]	1 month	121803.9 nmol/L	(9)
	6 months	116634.3 nmol/L	(9)
	12 months	106172.8 nmol/L	(9)
TG O-52:2 [NL-18:1]	1 month	129622.4 nmol/L	(9)
	6 months	112779.7 nmol/L	(9)
	12 months	113884.5 nmol/L	(9)
TG O-52:2 [NL-O-16:0]	1 month	5334.9 nmol/L	(9)
	6 months	4310.3 nmol/L	(9)
	12 months	4583.0 nmol/L	(9)
TG O-52:2 [NL-O-18:0]	1 month	3173.5 nmol/L	(9)
	6 months	3057.3 nmol/L	(9)
	12 months	2993.0 nmol/L	(9)
TG O-52:2 [NL-O-18:1]	1 month	23361.6 nmol/L	(9)
	6 months	21218.4 nmol/L	(9)
	12 months	20256.1 nmol/L	(9)

Unsp. = unspecified or non-specific

Supplementary Table 2 (continued). TG(O)s reportedly present in human milk samples

TG(O) species/molecular species	Timepoint of sample collection	Concentration/proportion	Ref.
TG O-52:3	1 month	4880.1 nmol/L	(9)
	6 months	3995.7 nmol/L	(9)
	12 months	4317.5 nmol/L	(9)
	Pooled 6 weeks, 3 months, 6 months	*Unsp.*	(4)
	Unsp.	11.3%	(8)
TG O-16:0_18:1_18:2	Pooled 6 weeks, 3 months, 6 months	*Unsp.*	(4)
TG O-18:1_16:0_18:2	Pooled 6 weeks, 3 months, 6 months	*Unsp.*	(4)
TG O-18:2_16:0_18:1	Pooled 6 weeks, 3 months, 6 months	*Unsp.*	(4)
TG O-16:0/18:2/18:1	*Unsp.*	*Unsp.*	(8)
TG O-18:1/16:0/18:2	*Unsp.*	*Unsp.*	(8)
TG O-18:2/16:0/18:1	*Unsp.*	*Unsp.*	(8)
TG O-52:3 [NL-16:0]	1 month	5662.0 nmol/L	(9)
	6 months	4675.4 nmol/L	(9)
	12 months	4657.3 nmol/L	(9)
TG O-52:3 [NL-18:1]	1 month	4355.9 nmol/L	(9)
	6 months	3439.2 nmol/L	(9)
	12 months	3499.4 nmol/L	(9)

Unsp. = unspecified or non-specific

Supplementary Table 2 (continued). TG(O)s reportedly present in human milk samples

TG(O) species/molecular species	Timepoint of sample collection	Concentration/proportion	Ref.
TG O-52:3 [NL-18:3]	1 month	91.7 nmol/L	(9)
	6 months	81.8 nmol/L	(9)
	12 months	88.1 nmol/L	(9)
TG O-52:4	1 month	4427.9 nmol/L	(9)
	6 months	4308.4 nmol/L	(9)
	12 months	4381.0 nmol/L	(9)
TG O-52:4 [NL-16:0]	1 month	85.2 nmol/L	(9)
	6 months	77.3 nmol/L	(9)
	12 months	69.3 nmol/L	(9)
TG O-52:4 [NL-18:1]	1 month	47.4 nmol/L	(9)
	6 months	33.8 nmol/L	(9)
	12 months	33.4 nmol/L	(9)
TG O-52:4 [NL-18:2]	1 month	156.5 nmol/L	(9)
	6 months	133.2 nmol/L	(9)
	12 months	133.3 nmol/L	(9)
TG O-52:4 [NL-18:3]	1 month	70.8 nmol/L	(9)
	6 months	52.2 nmol/L	(9)
	12 months	53.4 nmol/L	(9)
	12 months	3499.4 nmol/L	(9)

Unsp. = unspecified or non-specific

Supplementary Table 2 (continued). TG(O)s reportedly present in human milk samples

TG(O) species/molecular species	Timepoint of sample collection	Concentration/proportion	Ref.
TG O-54:0	1 month	220.8 nmol/L	(9)
	6 months	181.5 nmol/L	(9)
	12 months	158.1 nmol/L	(9)
	Pooled 6 weeks, 3 months, 6 months	*Unsp.*	(4)
TG O-16:0_16:0_22:0	Pooled 6 weeks, 3 months, 6 months	*Unsp.*	(4)
TG O-16:0_18:0_20:0	Pooled 3 months, 6 months	*Unsp.*	(4)
TG O-18:0_16:0_20:0	Pooled 6 weeks, 3 months, 6 months	*Unsp.*	(4)
TG O-20:0_16:0_18:0	Pooled 6 weeks, 3 months, 6 months	*Unsp.*	(4)
TG O-22:0_16:0_16:0	Pooled 6 weeks, 3 months, 6 months	*Unsp.*	(4)
TG O-54:0 [NL-18:0]	1 month	368.8 nmol/L	(9)
	6 months	276.8 nmol/L	(9)
	12 months	202.6 nmol/L	(9)
TG O-54:1	1 month	2780.2 nmol/L	(9)
	6 months	2296.6 nmol/L	(9)
	12 months	1982.1 nmol/L	(9)
	Pooled 6 weeks, 3 months, 6 months	*Unsp.*	(4)
	Unsp.	2.7%	(8)
TG O-16:0_18:1_20:0	Pooled 6 weeks, 3 months, 6 months	*Unsp.*	(4)
TG O-18:0_18:0_18:1	Pooled 6 weeks, 3 months, 6 months	*Unsp.*	(4)

Unsp. = unspecified or non-specific

Supplementary Table 2 (continued). TG(O)s reportedly present in human milk samples

TG(O) species/molecular species	Timepoint of sample collection	Concentration/proportion	Ref.
TG O-20:0_16:0_18:1	Pooled 6 weeks, 3 months, 6 months	*Unsp.*	(4)
TG O-16:0/18:1/20:0	*Unsp.*	*Unsp.*	(8)
TG O-18:0/18:1/18:0	*Unsp.*	*Unsp.*	(8)
TG O-20:0/18:1/16:0	*Unsp.*	*Unsp.*	(8)
TG O-54:1 [NL-18:0]	1 month	4171.7 nmol/L	(9)
	6 months	3620.5 nmol/L	(9)
	12 months	2722.3 nmol/L	(9)
TG O-54:1 [NL-18:1]	1 month	5815.0 nmol/L	(9)
	6 months	4782.3 nmol/L	(9)
	12 months	3826.0 nmol/L	(9)
TG O-54:2	1 month	12856.2 nmol/L	(9)
	6 months	10960.1 nmol/L	(9)
	12 months	9141.2 nmol/L	(9)
	Multiple timepoints	*Unsp.*	(4, 10)
	Unsp.	8.2%	(8)
TG O-18:0_18:1_18:1	Pooled 6 weeks, 3 months, 6 months	*Unsp.*	(4)
TG O-18:1_18:0_18:1	Pooled 6 weeks, 3 months, 6 months	*Unsp.*	(4)
TG O-16:0/18:1/20:1	*Unsp.*	*Unsp.*	(8)
TG O-16:0/18:2/20:0	*Unsp.*	*Unsp.*	(8)

Unsp. = unspecified or non-specific

Supplementary Table 2 (continued). TG(O)s reportedly present in human milk samples

TG(O) species/molecular species	Timepoint of sample collection	Concentration/proportion	Ref.
TG O-18:0/18:2/18:0	*Unsp.*	*Unsp.*	(8)
TG O-18:1/16:0/20:1	*Unsp.*	*Unsp.*	(8)
TG O-18:1/18:1/18:0	*Unsp.*	*Unsp.*	(8)
TG O-54:2 [NL-18:1]	1 month	55529.0 nmol/L	(9)
	6 months	47195.1 nmol/L	(9)
	12 months	35581.8 nmol/L	(9)
TG O-54:2 [NL-O-16:0]	1 month	1220.5 nmol/L	(9)
	6 months	917.8 nmol/L	(9)
	12 months	909.2 nmol/L	(9)
TG O-54:2 [NL-O-18:0]	1 month	5999.7 nmol/L	(9)
	6 months	5374.2 nmol/L	(9)
	12 months	4047.6 nmol/L	(9)
TG O-54:2 [NL-O-18:1]	1 month	1714.4 nmol/L	(9)
	6 months	1573.2 nmol/L	(9)
	12 months	1234.3 nmol/L	(9)

Unsp. = unspecified or non-specific

Supplementary Table 2 (continued). TG(O)s reportedly present in human milk samples

TG(O) species/molecular species	Timepoint of sample collection	Concentration/proportion	Ref.
TG O-54:3	1 month	10040.9 nmol/L	(9)
	6 months	8852.1 nmol/L	(9)
	12 months	7864.0 nmol/L	(9)
	Multiple timepoints	*Unsp.*	(4, 10)
	Unsp.	9.6%	(8)
TG O-18:0_18:1_18:2	Pooled 6 weeks, 3 months, 6 months	*Unsp.*	(4)
TG O-18:1_18:1_18:1	Pooled 6 weeks, 3 months, 6 months	*Unsp.*	(4)
TG O-16:0/18:2/20:1	*Unsp.*	*Unsp.*	(8)
TG O-16:0/18:1/20:2	*Unsp.*	*Unsp.*	(8)
TG O-18:0/18:1/18:2	*Unsp.*	*Unsp.*	(8)
TG O-18:1/18:2/18:0	*Unsp.*	*Unsp.*	(8)
TG O-54:3 [NL-18:1]	1 month	38569.0 nmol/L	(9)
	6 months	33575.8 nmol/L	(9)
	12 months	29444.7 nmol/L	(9)
TG O-54:3 [NL-O-16:0]	1 month	383.1 nmol/L	(9)
	6 months	257.5 nmol/L	(9)
	12 months	313.5 nmol/L	(9)

Unsp. = unspecified or non-specific

Supplementary Table 2 (continued). TG(O)s reportedly present in human milk samples

TG(O) species/molecular species	Timepoint of sample collection	Concentration/proportion	Ref.
TG O-54:3 [NL-O-18:0]	1 month	1601.3 nmol/L	(9)
	6 months	1488.0 nmol/L	(9)
	12 months	1383.3 nmol/L	(9)
TG O-54:3 [NL-O-18:1]	1 month	4619.4 nmol/L	(9)
	6 months	4004.6 nmol/L	(9)
	12 months	3499.1 nmol/L	(9)
TG O-54:4	1 month	1711.1 nmol/L	(9)
	6 months	1486.0 nmol/L	(9)
	12 months	1635.1 nmol/L	(9)
	Unsp.	5.8%	(8)
TG O-18:1/18:2/18:1	*Unsp.*	5.8%	(8)
TG O-54:4 [NL-O-16:0]	Multiple timepoints	*Unsp.*	(9)
TG O-54:4 [NL-O-18:0]	Multiple timepoints	*Unsp.*	(9)
TG O-54:4 [NL-18:1]	1 month	1712.0 nmol/L	(9)
	6 months	1366.1 nmol/L	(9)
	12 months	1397.9 nmol/L	(9)
TG O-54:4 [NL-18:2]	1 month	1479.0 nmol/L	(9)
	6 months	1254.5 nmol/L	(9)
	12 months	1285.4 nmol/L	(9)

Unsp. = unspecified or non-specific

Supplementary Table 2 (continued). TG(O)s reportedly present in human milk samples

TG(O) species/molecular species	Timepoint of sample collection	Concentration/proportion	Ref.
TG O-54:4 [NL-O-18:1]	1 month	365.3 nmol/L	(9)
	6 months	319.6 nmol/L	(9)
	12 months	323.1 nmol/L	(9)
TG O-56:0	Pooled 6 weeks, 3 months, 6 months	*Unsp.*	(4)
TG O-18:0_16:0_22:0	Pooled 6 weeks, 3 months, 6 months	*Unsp.*	(4)
TG O-22:0_16:0_18:0	Pooled 6 weeks, 3 months, 6 months	*Unsp.*	(4)
TG O-24:0_16:0_16:0	Pooled 6 weeks, 3 months, 6 months	*Unsp.*	(4)
TG O-56:1	Pooled 6 weeks, 3 months, 6 months	*Unsp.*	(4)
TG O-16:0_18:1_22:0	Pooled 6 weeks, 3 months, 6 months	*Unsp.*	(4)
TG O-18:0_18:1_20:0	Pooled 6 weeks, 3 months, 6 months	*Unsp.*	(4)
TG O-18:1_16:0_22:0	Pooled 6 weeks, 3 months, 6 months	*Unsp.*	(4)
TG O-22:0_16:0_18:1	Pooled 6 weeks, 3 months, 6 months	*Unsp.*	(4)
TG O-56:2	Pooled 6 weeks, 3 months, 6 months	*Unsp.*	(4)
	Unsp.	2.3%	(8)
TG O-16:0_18:1_22:1	Pooled 6 weeks, 3 months, 6 months	*Unsp.*	(4)
TG O-16:0/18:1/22:1	*Unsp.*	*Unsp.*	(8)
TG O-18:0/18:1/20:1	*Unsp.*	*Unsp.*	(8)
TG O-18:1/18:1/20:0	*Unsp.*	*Unsp.*	(8)
TG O-56:3	Pooled 6 weeks, 3 months, 6 months	*Unsp.*	(4)

Unsp. = unspecified or non-specific

Supplementary Table 2 (continued). TG(O)s reportedly present in human milk samples

TG(O) species/molecular species	Timepoint of sample collection	Concentration/proportion	Ref.
TG O-16:0_18:1_22:2	Pooled 6 weeks, 3 months, 6 months	*Unsp.*	(4)
TG O-58:2	Pooled 6 weeks, 3 months, 6 months	*Unsp.*	(4)
TG O-24:1_16:0_18:1	Pooled 6 weeks, 3 months, 6 months	*Unsp.*	(4)
TG O-58:3	Pooled 6 weeks, 3 months, 6 months	*Unsp.*	(4)

Unsp. = unspecified or non-specific

1. Sawane K, Takahashi I, Ishikuro M, Takumi H, Orui M, Noda A, et al. Exploring the association between human breast milk lipids and early adiposity rebound in children: a case-control study. Nutrition. 2025;135:112739. DOI: https://doi.org/10.1016/j.nut.2025.112739.
2. Zhao Y-c, Zhang Y, Liu C-x, Yan D-y, Dong P. Compositional Differences between Preterm Milk of Different Gestational Ages with the Term Milk: A Comparative Lipidomic Study by LC-MS/MS. Eur J Lipid Sci Technol. 2022;124(7):2100224. DOI: https://doi.org/10.1002/ejlt.202100224.
3. Zhong H, Zhang J, Xia J, Zhu Y, Chen C, Shan C, et al. Influence of gestational diabetes mellitus on lipid signatures in breast milk and association with fetal physical development. Front Nutr. 2022;Volume 9 - 2022. DOI: https://doi.org/10.3389/fnut.2022.924301.
4. Lee MLX, Nguyen KNT, Pang WW, Torta F, Chong YS, Eriksson JG, et al. Measurement of Endogenous Fatty Acid Esters of Hydroxy Fatty Acid (FAHFA) and Alkyl-diacylglycerol (TG(O)) from Human Breast Milk. ACS Pharmacology & Translational Science. 2026:101-13. DOI: https://doi.org/10.1021/acsptsci.5c00601.
5. Olajide TM, Li T, Shu L, Cao H, Li Y, Cao W. Analysis of fatty acid esters of hydroxy fatty acid in human milk using chemical isotope labeling-assisted LC-MS. Food Chem. 2025;492:145480. DOI: https://doi.org/10.1016/j.foodchem.2025.145480.
6. Brejchova K, Paluchova V, Brezinova M, Cajka T, Balas L, Durand T, et al. Triacylglycerols containing branched palmitic acid ester of hydroxystearic acid (PAHSA) are present in the breast milk and hydrolyzed by carboxyl ester lipase. Food Chem. 2022;388:132983. DOI: https://doi.org/10.1016/j.foodchem.2022.132983.
7. Dong P, Zhang Y, Liu C-x, Yan D-y, Xu X, Li A-h, et al. Association between Human Milk Fatty Acid Esters of Hydroxy Fatty Acids and Maternal Body Mass Index and Early Infant Growth: A Longitudinal Study. J Nutr. 2025;155(7):2290-9. DOI: https://doi.org/10.1016/j.tjnut.2025.05.022.
8. Wang H, Zhao Y, Wu T, Hou Y, Chen X, Shi J, et al. Development and application of a pseudotargeted lipidomics method for alkylglycerol analysis. Food Chem. 2024;437:137926. DOI: https://doi.org/10.1016/j.foodchem.2023.137926.
9. George AD, Paul S, Wang T, Huynh K, Giles C, Mellett N, et al. Defining the lipid profiles of human milk, infant formula, and animal milk: implications for infant feeding. Front Nutr. 2023;10:1227340. DOI: https://doi.org/10.3389/fnut.2023.1227340.
10. Hewelt-Belka W, Garwolińska D, Młynarczyk M, Kot-Wasik A. Comparative Lipidomic Study of Human Milk from Different Lactation Stages and Milk Formulas. Nutrients. 2020;12(7):2165. DOI: https://doi.org/10.3390/nu12072165.
